# Synthesis and Biological Activity Assessment of Caffeic Acid Azaheterocyclic Amide Derivatives

**DOI:** 10.3390/ijms27188262

**Published:** 2026-09-16

**Authors:** Yang Xu, Hong Shen, Youxia Liu, Yuan Cao, Jingjing Huang, Pu Yan, Yongbiao Wei, Zhenwen Zhao, Xiangjun Liu, Dihua Shangguan

**Affiliations:** 1 Beijing National Laboratory for Molecular Sciences, Key Laboratory of Analytical Chemistry for Living Biosystems, CAS Research/Education Center for Excellence in Molecular Sciences, Institute of Chemistry, Chinese Academy of Sciences, Beijing 100190, China; xuyang22@mails.ucas.ac.cn (Y.X.); ggq-eovyd79pd@dingtalk.com (J.H.);; 2 School of Chemical Sciences, University of Chinese Academy of Sciences, Beijing 100049, China; 3 School of Molecular Medicine, Hangzhou Institute for Advanced Study, University of Chinese Academy of Sciences, Hangzhou 310013, China; 4 Department of Otolaryngology, Head and Neck Surgery, Peking University First Hospital, Beijing 100034, China; shen_ent@163.com; 5 Department of Pharmaceutical Chemistry, School of Pharmaceutical Sciences, Guangxi Medical University, No. 22, Shuangyong Road, Nanning 530021, China

**Keywords:** caffeic acid amide derivatives, N-heterocycle, antitumor activity, antioxidant, anti-inflammatory, neuroprotection

## Abstract

Natural caffeic acid derivatives are ubiquitous plant phenolic compounds with documented antitumor, antioxidant, and anti-inflammatory bioactivities. However, their clinical application is limited by low bioactivity potency and poor stability. Therefore, rational design and synthesis of novel caffeic acid derivatives are critical to improve their druglikeness and broaden biomedical applications. Herein, fourteen amide derivatives (**H1**–**H14**) were synthesized by conjugating three 3,4-substituted caffeic acid skeletons with five nitrogen-containing heterocycles. Bioactivity evaluation revealed that these derivatives exhibited structure- and cell-dependent biological profiles. Among them, compounds **H8** and **H13** containing 5-methoxytryptamine exerted the most potent antitumor activity by suppressing DNA and RNA synthesis and arresting the cell cycle at the G2/M phase. Derivatives with a catechol moiety (**H11**–**H14**) possessed prominent antioxidant activity, while **H11** and **H12**, containing morpholine and 1-methylpiperazine respectively, exhibited remarkable anti-inflammatory activity. Compound **H5** also presented significant protective effects against H_2_O_2_-induced neuronal injury. The cellular uptake assay revealed a correlation with clogP values: derivatives with clogP > 2.5 showed higher cellular internalization. Collectively, nitrogen-containing heterocyclic moieties and 3,4-substitution effectively modulate the multiple bioactivities of caffeic acid derivatives, providing valuable guidance for further structural optimization and drug development.

## 1. Introduction

Caffeic acid is a natural hydroxycinnamic acid compound, which is widely present in various dietary sources, especially abundant in coffee, apples, cherries, plums, kiwis and blueberries [1]. Its molecular backbone features a catechol moiety bearing two phenolic hydroxyl groups at the C3 and C4 positions; the catechol moiety is susceptible to oxidation and readily converts into ortho-quinone [2,3]. Such redox property plays a critical role in the various biological activities of caffeic acid. It plays important roles in plant growth, such as protecting plants from ultraviolet damage, inhibiting bacterial growth, and so on [4,5]. Moreover, numerous studies have explored its pharmacological activities and demonstrated that caffeic acid exhibits antioxidant, anti-inflammatory, antibacterial, antihypertensive, hypoglycemic, and antitumor activities [6,7,8]. For example, in the field of antioxidation, it not only scavenges harmful free radicals and reactive oxygen species via phenolic hydroxyl groups to alleviate oxidative stress, but also blocks transition metal ion-induced lipid peroxidation through metal chelation [9,10]. In the field of antitumor research, caffeic acid can inhibit key signaling pathways, such as phosphatidylinositol 3-kinase/protein kinase B (PI3K/Akt), mitogen-activated protein kinase (MAPK), and nuclear factor kappa B (NF-κB), thereby downregulating the expression of vascular endothelial growth factor and suppressing tumor angiogenesis [11,12,13,14]. Meanwhile, it directly inhibits the proliferation of cancer cells and may exert synergistic effects with chemotherapeutic agents, enhancing antitumor efficacy or reversing drug resistance [11]. However, the weak bioactivity and insufficient stability of caffeic acid limit its applications both in vitro and in vivo [15,16,17].

Natural caffeic acid derivatives possess broad-spectrum biological activities and are ubiquitously present in various plant species, thereby attracting extensive research attention. Caffeic acid phenethyl ester (CAPE), a well-known natural derivative of caffeic acid obtained from propolis, has been extensively studied, and has exhibited excellent properties in terms of antioxidant, antimicrobial, anti-inflammatory, and anticancer activities [18]. Studies have shown that CAPE can act on multiple biological pathways and affect the expression of various transcription factors, including cyclooxygenase-2 (COX-2), hypoxia-inducible factor-1α (HIF-1α), interleukin-6 (IL-6), and tumor necrosis factor-α (TNF-α), thereby exerting a variety of biological functions [19,20]. Chlorogenic acid, the most widely distributed and abundant derivative of caffeic acid in nature, can activate the nuclear factor erythroid 2-related factor 2 (Nrf2) pathway, inhibit inflammatory pathways such as NF-κB and TLR4, downregulate VEGF, and suppress LDL oxidation [21,22,23]. It exhibits excellent properties in antioxidant, anti-inflammatory, antitumor and cardiovascular protective properties. While natural caffeic acid derivatives display a wide spectrum of biological activities, their generally weak bioactivity precludes most of them from being developed as standalone drugs for clinical use.

Therefore, to achieve better properties, many caffeic acid derivatives have been synthesized, mainly including caffeic acid esters, caffeic acid amides, and caffeic acid hybrids [24,25]. For example, De Vita et al. synthesized a group of caffeic acid ester derivatives via esterification, among which compounds **1f**, **1g** and **1i** exhibited higher activity [26]. Wang et al. synthesized twelve N-hydroxycinnamoyl amino acid amide ethyl esters using amino acids and caffeic acid homologs as starting materials, and the results showed that compound **3k** exhibited the strongest free radical scavenging activity [27]. Peng et al. prepared several hybrids by linking caffeic acid with sulfonamides, and the results showed that these hybrids exhibited favorable effects in antioxidant, anticoagulant and antibacterial activities [28,29].

Nitrogen-containing heterocycles, such as pyrrole, imidazole, and pyrazole, are widely found in nature and frequently present in natural products and pharmaceutical molecules. They possess diverse pharmacological properties, such as anti-inflammatory, antibacterial and anticancer activities [30,31]. Notably, several bioactive compounds mentioned above also incorporate heterocyclic moieties like furan and pyrimidine, suggesting that heterocyclic moieties can profoundly modulate the biological activity of candidate compounds. In addition, the amide group is an important structural unit in biological molecules. For instance, peptide bonds in proteins are formed by amide linkages, which endow amide compounds with natural affinity and biocompatibility in living organisms. As a result, a large number of pharmaceutical agents contain amide groups [32].

Herein, five nitrogen-containing heterocyclic compounds, namely morpholine, 1-methylpiperazine, 5-methoxytryptamine, 1-(2-aminoethyl)piperidine and 2-aminoimidazole, were selected as raw materials. By coupling these five nitrogen heterocycles separately with the carboxyl groups of three caffeic acid derivatives bearing different substituents at the 3,4 positions, a total of 14 caffeic acid amide derivatives were designed and synthesized. They were comprehensively investigated and evaluated for several key biological activities, including antitumor, antioxidant, anti-inflammatory, neuroprotective, and antibacterial activities. The structure–activity relationships (SARs) and cellular uptake capacities of these compounds were further investigated.

## 2. Results and Discussion

### 2.1. Synthesis of Compounds

**Scheme 1:**
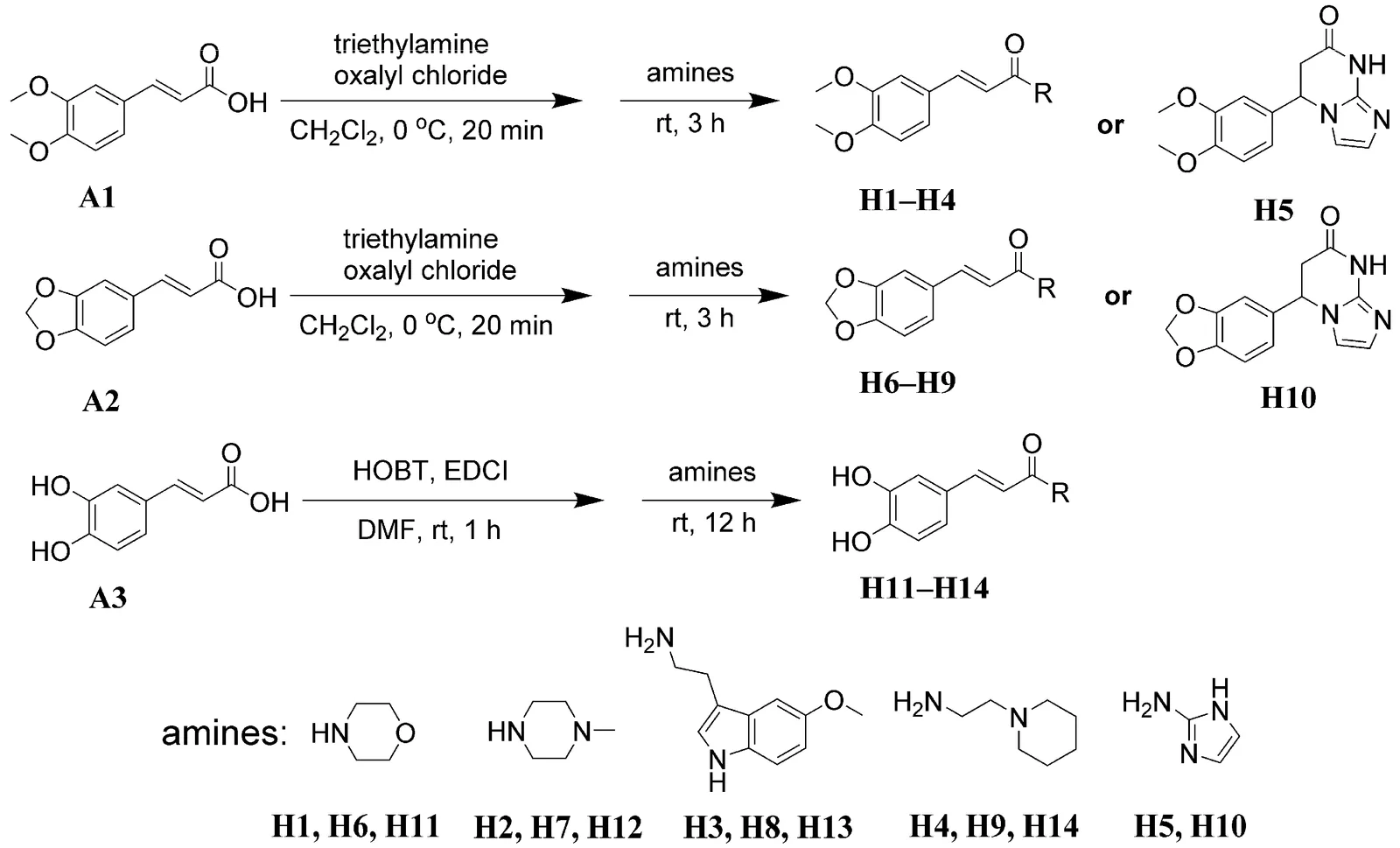
Synthetic routes of caffeic acid amide derivatives.

The synthetic route is illustrated in Scheme 1. Briefly, 3,4-Dimethoxycinnamic acid (**A1**), 3,4-methylenedioxycinnamic acid (**A2**), and caffeic acid (**A3**) were employed as the starting materials. Each was subjected to amidation with morpholine, 1-methylpiperazine, 5-methoxytryptamine, 1-(2-aminoethyl) piperidine and 2-aminoimidazole, yielding 14 caffeic acid amide derivatives designated as **H1**–**H14**. Among them, **H1**–**H10** were synthesized via the acyl chloride method [33], with isolated yields ranging from 52–80%, while **H11**–**H14** were synthesized using the EDCI/HOBT condensation [34], affording yields of 22–61%. Notably, **H5** and **H10** do not correspond to simple amidation products, but rather cyclized species arising from further intramolecular Michael addition. All compounds were purified by silica gel column chromatography. Several factors account for the low yields of **H12**–**H14**: (i) phenolic hydroxyl groups are susceptible to side reactions, including oxidation; and (ii) partial adsorption of products on silica gel, which causes material loss during column chromatography. The yields (%) of each synthesized compound have been listed in the Experimental Section. All target structures were fully characterized by ^1^H NMR, ^13^C NMR, and HR-MS(ESI) (see the Supporting Information). Among these compounds, **H3**, **H4**, **H5**, **H8**, **H9**, **H10** and **H14** are newly reported compounds. Although other compounds have been previously reported [34,35,36,37], their biological activities have not been comprehensively investigated. Therefore, in the present study, we systematically evaluated the biological activities of all synthesized compounds.

### 2.2. Antiproliferative Activity

**Figure 1:**
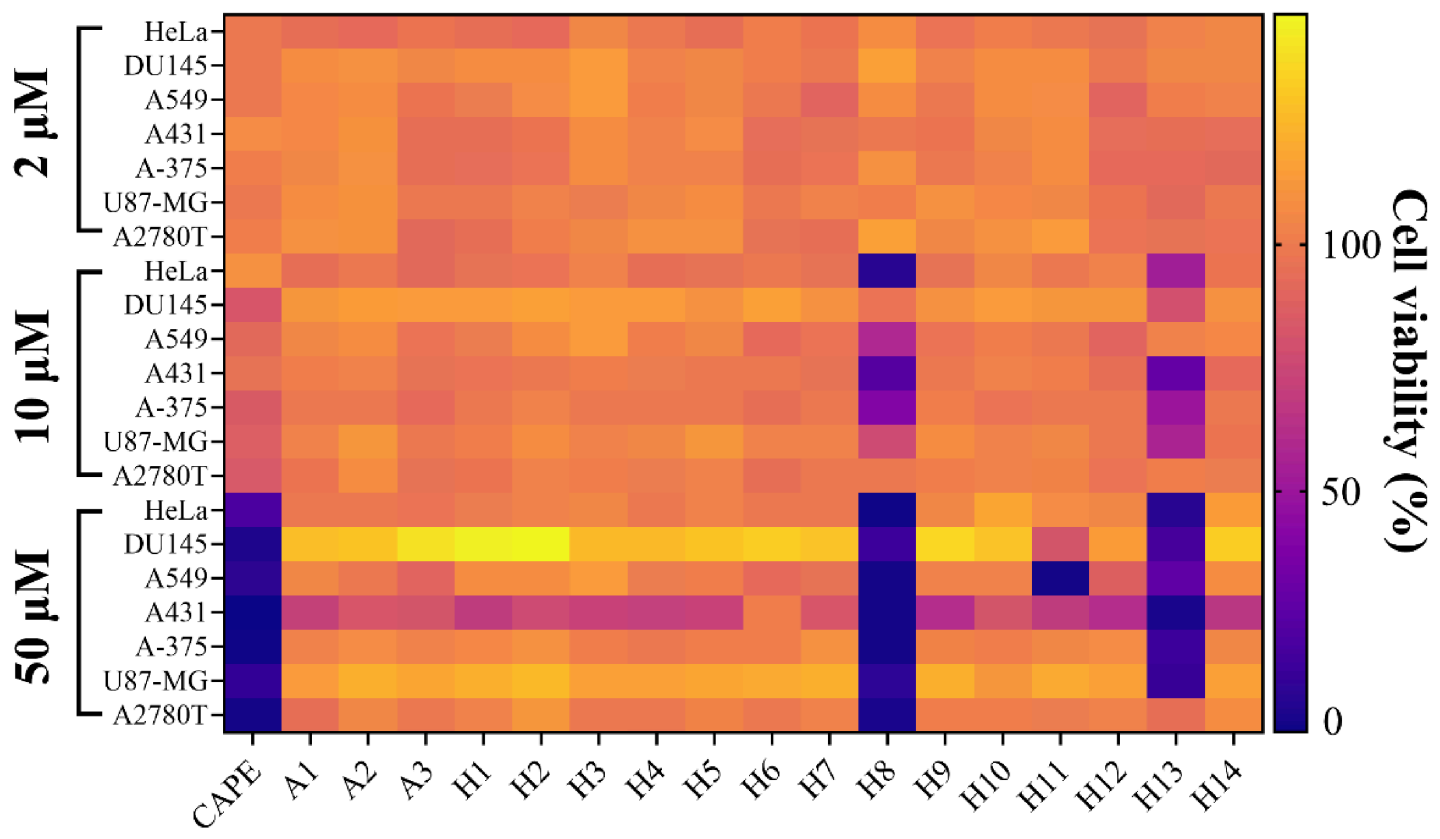
Relative cell viabilities of different cell lines treated with diverse compounds at varying concentrations (2, 10, 50 μM, *n* = 3) for 120 h.

The in vitro antiproliferative activities of the fourteen synthesized compounds, three starting materials (3,4-dimethoxycinnamic acid (**A1**), 3,4-methylenedioxycinnamic acid (**A2**), and caffeic acid (**A3**)), and the natural derivative **CAPE** were evaluated by the CCK-8 assay. Cells including HeLa, DU145, A549, A431, A-375, U87-MG, and A2780T were incubated with the tested compounds at concentrations of 2, 10 and 50 μM for 120 h. As shown in Figure 1, at 50 μM, **CAPE** significantly inhibited the proliferation of all tested cell lines, whereas **A1**, **A2**, and **A3** exhibited almost no antiproliferative activity. Among the synthetic derivatives, inhibitory effects varied markedly in a cell-line-dependent manner. H8 and H13 displayed superior antiproliferative activity against most tumor cells at 50 μM. Notably, both H8 and H13 bear a methoxytryptamine fragment, implying that this heterocyclic moiety contributes substantially to antitumor potency.

Subsequently, to quantitatively compare the bioactivity, the half-maximal inhibitory concentrations (IC_50_) of the two potent antiproliferative candidates, **H8** and **H13**, and **CAPE** were further determined against a panel of cell lines including HeLa, A431, A-375, THP-1, MKN-45, A2780T, BeWo, BC-1, LX-2, K562, LoVo, RAW264.7, and L-02 cells following 48 h treatment. As summarized in Table 1, **CAPE**, **H8** and **H13** exhibited distinct cell-type-dependent antiproliferative potency against the tested cell lines. Overall, compound **H8** displayed broad-spectrum and superior inhibitory effects across most tumor cell lines, showing far lower IC_50_ values than **CAPE** in the majority of cancer cells, which indicated that structural modification greatly improved the antitumor activity. In contrast, **H13** exerted selective cytotoxicity rather than universal growth suppression. **CAPE** presented moderate antitumor activity toward most tumor cells, but possessed extremely potent inhibitory activity against RAW264.7 macrophages. Individually, **H8** displayed excellent efficacy against HeLa, A-375 and K562 cells with IC_50_ values below 10 μM, representing the most promising antitumor candidate. **H13** showed excellent activity only against EBC-1 cells, while it was nearly inactive toward A2780T cells with an IC_50_ over 100 μM. Notably, **CAPE** exerted the strongest inhibitory effect on RAW264.7 cells, whereas **H8** showed weak activity against this cell line, revealing obvious target selectivity differences among these three compounds. In addition, **CAPE** failed to inhibit the proliferation of MKN-45 and EBC-1 cells, while both **H8** and **H13** effectively suppressed these two cell lines. Notably, H8 markedly suppressed the proliferation of L-02 normal hepatocytes. By contrast, CAPE and H13 displayed low proliferative inhibition, indicative of a more favorable therapeutic window for these two compounds. This observation highlights that nitrogen heterocyclic conjugation can broaden the antitumor spectrum and enhance bioactivity.

The superior anti-proliferative activity of **H8** against most cell lines compared with **H13** can be attributed to their molecular structure differences. The methylenedioxy group in **H8** endows it with higher lipophilicity than the ortho-dihydroxy group in **H13**, which facilitates cell-membrane penetration and contributes to enhanced cytotoxicity. Given the marked differences in biological activities between **H8** and **H13**, A-375 cells were chosen for further mechanistic investigations.

### 2.3. Effects of **H8** and **H13** on Nucleic Acid Synthesis and Cell Cycle in A-375 Cells

**Figure 2:**
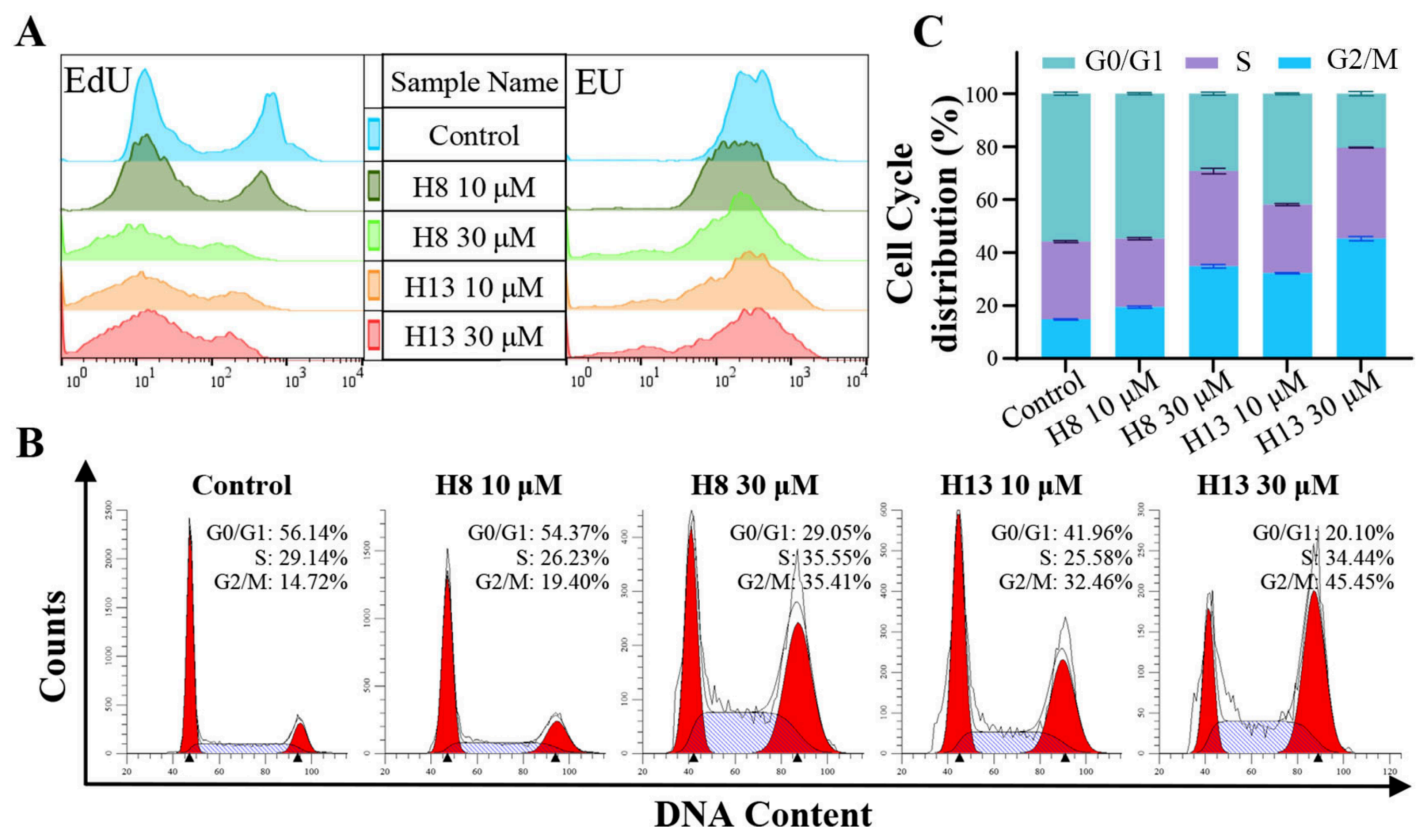
Antiproliferative mechanism study of H8 and H13. (**A**) Flow cytometry analysis of the incorporation of EdU (left) or EU (right) in A-375 cells after treatment with compound H8 or H13 for 24 h. (**B**) Cell cycle assay of A-375 cells treated with H8 or H13 for 24 h. (**C**) The percentage of cells in each cell cycle phase.

To elucidate the antiproliferative mechanism of **H8** and **H13** in A-375 cells, 5-Ethynyl-2′-deoxyuridine (EdU) and 5-ethynyluridine (EU) incorporation assays were performed to evaluate their effects on cellular nucleic acid synthesis. EdU and EU, as nucleoside analogs of thymidine and uridine, can be incorporated into newly synthesized DNA and RNA during cell proliferation, respectively. By conjugating EdU or EU with Alexa Fluor 647 fluorescent dye through click chemistry, quantitative analysis of cellular DNA and RNA synthesis can be realized. The results of the EdU incorporation assay showed that untreated A-375 cells exhibited a typical bimodal distribution: the low fluorescence intensity peak represented the background signal, and the high fluorescence intensity peak reflected active DNA synthesis (Figure 2A left). After treatment with 10 μM **H8** for 24 h, the proportion of cells with low fluorescence intensity increased, while the peak of high fluorescence intensity became lower and its corresponding area decreased. Treatment with 30 μM **H8**, 10 μM and 30 μM **H13** broadened the low-fluorescence peak with a remarkably enlarged area. Meanwhile, the high-fluorescence peak declined in height, shrank in area and exhibited distinctly reduced fluorescence intensity. These results indicate that treatment with **H8** and **H13** can markedly inhibit DNA replication. In the EU incorporation assay (Figure 2A right), untreated A-375 cells exhibited a single high-fluorescence peak. After treatment with 10 μM and 30 μM **H8** or **H13**, the peak fluorescence intensity slightly decreased and low-fluorescence cell populations emerged, suggesting that both compounds are capable of suppressing RNA synthesis.

To further explore the antiproliferative mechanism, cell cycle distribution was determined via flow cytometry. Treatment with **H8** and **H13** induced a dose-dependent G_2_/M phase arrest in A375 cells. Compared with the control group (G_2_/M phase: 14.72%), 10 μM H8 moderately elevated the G_2_/M fraction to 19.40%, and 30 μM H8 produced a pronounced increase to 35.41%. Notably, **H13** exhibited a more potent G_2_/M arrest effect: the proportion of G_2_/M phase cells reached 32.46% at 10 μM and further increased to 45.45% at 30 μM, accompanied by a sharp decrease in the G0/G1 phase population (Figure 2B,C). G_2_/M arrest is a well-documented antiproliferative mechanism that effectively blocks mitotic progression and suppresses tumor cell expansion. Our cell-cycle results are mechanistically consistent with the reduced DNA synthesis observed in EdU experiments and provide a plausible explanation for the cytostatic effects of **H8** and **H13**.

### 2.4. Antioxidant Activity

Caffeic acid has received much attention due to its remarkable antioxidant activity. The radical scavenging activities of the synthesized caffeic acid amide derivatives were determined by using the DPPH method and the ABTS method respectively. The results (Figure S1) showed that only the compounds containing a catechol moiety (**H11**–**H14**) exhibited significant free radical scavenging ability, and the scavenging rate increased in a concentration-dependent manner. In contrast, blocking the C3 and C4 phenolic hydroxyl groups via dimethoxy or methylenedioxy substitution led to a dramatic decline in antioxidant capacity. This phenomenon is closely related to the free radical scavenging mechanism of catechol groups: the vicinal phenolic hydroxyl groups readily donate hydrogen atoms to eliminate excess reactive oxygen species (ROS), which accounts for their strong antioxidant performance. Alkylation of phenolic hydroxyl groups eliminates hydrogen-donating sites and thus drastically weakens free radical scavenging ability. This result is consistent with previous literature reports, confirming that the catechol moiety is crucial for improving the antioxidant activity of these derivatives.

The EC_50_ values further quantified these differences (Table 2). **H11** and **H13** exhibited antioxidant efficacy comparable to that of caffeic acid (**A3**) and **CAPE**. By contrast, **H12** displayed weaker antioxidant capacity than **H11** and **H13**, and **H14** had the lowest activity among catechol-bearing derivatives. Such variation within **H11**–**H14** suggests that the attached heterocyclic fragment modulates antioxidant performance even when the intact catechol group is preserved. Heterocyclic moieties may alter molecular solubility, stability, or intermolecular interactions, which should be considered when designing caffeic-acid-based antioxidants.

### 2.5. Anti-Inflammatory Activity

**Figure 3:**
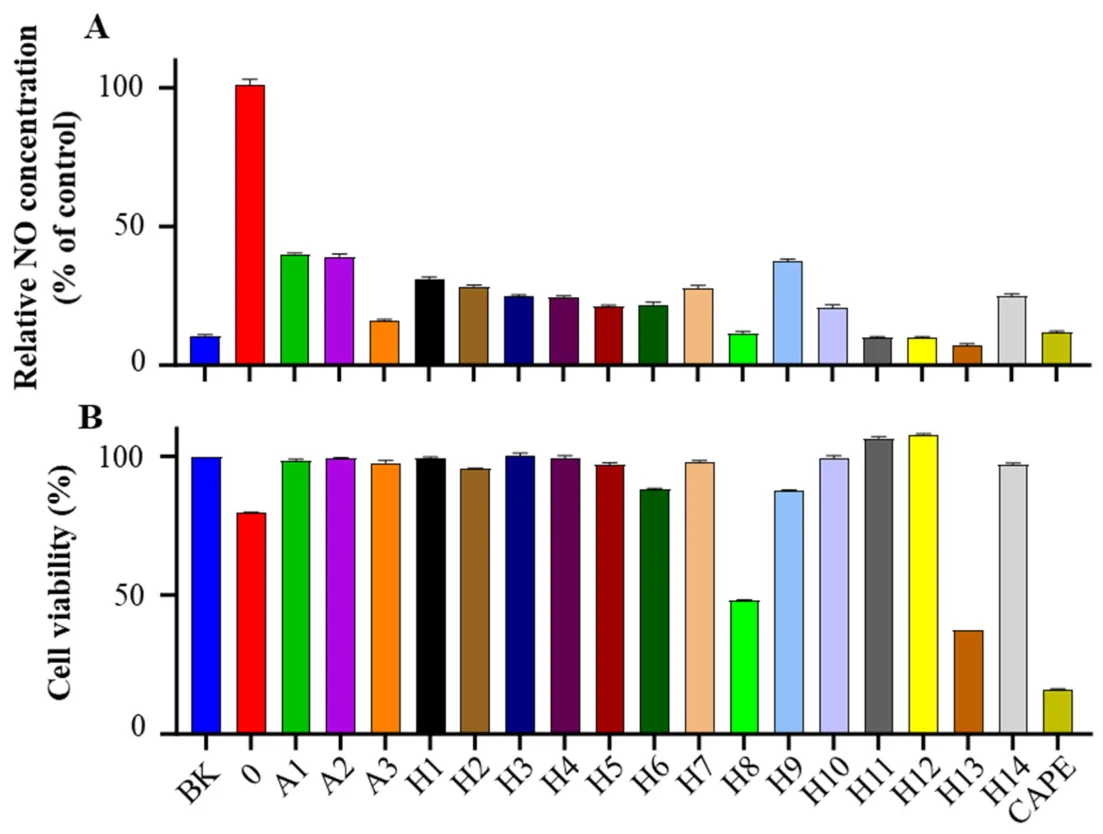
The anti-inflammatory and cytoprotective effects of compounds (100 μM) in RAW264.7 macrophages. (**A**) NO production in the culture medium of RAW264.7 cells following compound incubation and LPS stimulation for 48 h; (**B**) Cell viability of RAW264.7 cells after compound incubation and LPS stimulation for 48 h. BK: Blank control (without any treatment); 0: only LPS-treated control group.

As a crucial signaling molecule, nitric oxide (NO) is closely implicated in inflammation, and its level alterations reflect the immune activation state of cells [38]. Using lipopolysaccharide (LPS)-stimulated RAW264.7 mouse macrophages as an inflammatory model, the immunomodulatory activities of the synthesized caffeic acid derivatives were evaluated via NO release detection. As shown in Figure 3A, all compounds inhibited NO production. In particular, caffeic acid (**A3**), **H8**, **H11**, **H12**, **H13** and **CAPE** markedly inhibited NO release to a level similar to that of unstimulated cells, indicating their significant anti-inflammatory potential. However, cell viability assays revealed that **H8**, **H13**, and **CAPE** exhibited significant cytotoxicity at the same concentrations (Figure 3B). Accordingly, their NO-suppressive effect cannot be fully attributed to specific anti-inflammatory signaling inhibition and is partially confounded by reduced cell survival. In contrast, caffeic acid (**A3**), **H11**, **H12** and other tested compounds exhibited negligible cytotoxicity, and cell viability was significantly elevated relative to the LPS-treated control group, indicating their genuine intrinsic anti-inflammatory potential. IC_50_ values of **H11** and **H12** for inhibiting NO production were 11.91 ± 1.23 μM and 34.34 ± 9.39 μM respectively (Figure S2), highlighting their value as non-cytotoxic anti-inflammatory lead candidates. This phenotypic distinction stresses the necessity of combining NO quantification with parallel cytotoxicity testing for screening phenolic anti-inflammatory agents.

### 2.6. Neuroprotective Effect

**Figure 4:**
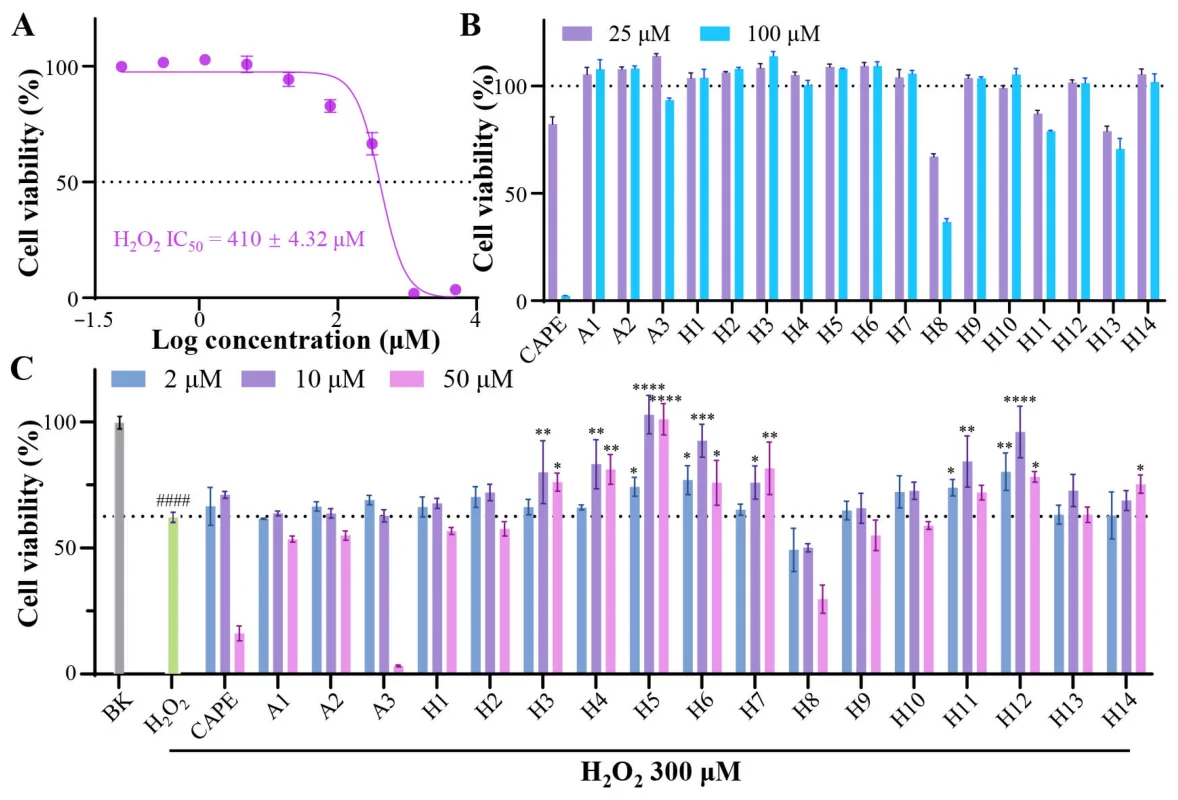
The compounds exert neuroprotective effects against H_2_O_2_-induced damage in SH-SY5Y cells. (**A**) Cell viability of SH-SY5Y cells after treatment with different concentrations of H_2_O_2_ for 48 h; (**B**) Cell viability of SH-SY5Y cells after treatment with 25 and 100 μM of different compounds for 48 h; (**C**) Cell viability of SH-SY5Y cells pretreated with various concentrations of compounds for 2 h followed by 48 h of H_2_O_2_ stimulation. Data are presented as mean ± SEM, *n* = 3. Compared with the control group (BK), the significance level of H_2_O_2_ treatment was #### *p* < 0.0001. Compared with treatment with only H_2_O_2_, the significance levels of the drug treatment groups were * *p* < 0.05, ** *p* < 0.01, *** *p* < 0.001, and **** *p* < 0.0001. BK: Blank control (without any treatment); H_2_O_2_: only H_2_O_2_-treated control group.

Alzheimer’s disease (AD) is a central nervous system degenerative disease characterized clinically by progressive memory loss and cognitive dysfunction [39]. In this study, an in vitro model of AD was established using the human neuroblastoma cell line SH-SY5Y, and H_2_O_2_-induced oxidative stress injury was applied to mimic the pathological process of AD. First, the cytotoxicity of H_2_O_2_ toward SH-SY5Y cells was evaluated. As shown in Figure 4A, cell viability decreased in a dose-dependent manner with increasing H_2_O_2_ concentration, with an IC_50_ value of 410 μM. Therefore, 300 μM H_2_O_2_ (corresponding to 66% cell viability) was chosen for subsequent experiments to ensure an adequate number of cells for further investigation.

Then, the antiproliferative potential of the synthesized caffeic acid amide derivatives (**H1**–**H14**), **CAPE**, **A1**, **A2**, and caffeic acid (**A3**) was further evaluated using SH-SY5Y cells. Figure 4B reveals that **CAPE**, **H8**, **H11**, and **H13** possessed moderate antiproliferative activity on SH-SY5Y cells at 100 μM, whereas other compounds had little influence on SH-SY5Y viability. Furthermore, to investigate the neuroprotective effects of these compounds, their influence on the viability of H_2_O_2_-treated SH-SY5Y cells was examined. As shown in Figure 4C, **H3**, **H4**, **H5**, **H6**, **H7**, **H11**, and **H12** were found to increase cell viability to varying degrees, with **H5** exhibiting the most significant neuroprotective effect, markedly reversing the reduction in cell viability induced by H_2_O_2_, suggesting its good neuroprotective capacity against H_2_O_2_-induced neuronal injury. These results demonstrate that certain caffeic-acid amide derivatives can defend neurons against oxidative insult and expand their pharmacological scope beyond antitumor and anti-inflammatory applications. It should be noted that neuroprotection was not strictly correlated with in-vitro radical-scavenging capacity, suggesting that neuroprotection may be another mechanism.

### 2.7. Antibacterial Activity

Caffeic acid and its derivatives have been widely reported to possess antibacterial activity. Herein, the antibacterial properties of the 14 synthesized caffeic acid amide derivatives, together with **CAPE**, **A1**, **A2**, and caffeic acid (**A3**), were evaluated against three Gram-negative and three Gram-positive bacterial strains via the broth microdilution assay. Their minimum inhibitory concentration (MIC) results showed that only **H13** and **CAPE** showed moderate antibacterial activity against certain strains. All other compounds displayed weak inhibitory effects, with their MIC values exceeding 256 μg/mL against all tested bacterial strains (Table S1). Although natural caffeic acid is reported to possess mild antibacterial activity, our nitrogen-containing heterocyclic derivatization strategy mainly enhanced antitumor, antioxidant, anti-inflammatory and neuroprotective properties without substantially improving antibacterial potency.

### 2.8. The Influence of Compound Structure on Cellular Uptake

The above experimental results indicate that the synthesized derivatives exert varying degrees of inhibitory activity against multiple tumor cell lines in vitro. It is worth noting that compounds bearing similar functional groups still differ substantially in cytotoxicity. Taking A-375 cells as an example, the IC_50_ values of compounds **H3**, **H8** and **H13** differ by 2.7 to 20-fold. Such distinct activity disparities are likely closely associated with compound structural features: molecular structure may influence membrane permeability and cellular uptake, which ultimately determine their cytotoxic potency. In addition, the Calculated Octanol–Water Partition Coefficient (cLogP), which reflects the lipophilicity of a compound, is a key physicochemical parameter for evaluating its drug-likeness and is widely used in drug design.

To investigate the cellular uptake of the compounds and its correlation with their cytotoxicity, an LC-MS method was established to quantify the amount of compounds taken up by cells. The compounds (**A1**–**A3**, **H1**–**H14**, 100 µM each) and the internal standard N-p-trans-coumaroyltyramine (NPC, 100 µM) were individually co-incubated with A-375 and RAW 264.7 cells for 1 h, respectively. Subsequently, the intracellular content ratio of each compound to NPC (Cs/Cn) was used to compare their cellular uptake efficiencies. The cLogP values of all synthesized compounds were listed in Table S2. Among derivatives bearing the same heterocyclic moiety, the 3,4-methylenedioxy-substituted analogs exhibit higher cLogP values than those with 3,4-dimethoxy substitution, which in turn show greater cLogP values than catechol-substituted derivatives. For derivatives with identical 3,4-substituents, the cLogP values of analogs containing different heterocycles follow the order below: 5-methoxytryptamine > 1-(2-aminoethyl)piperidine > 2-aminoimidazole ≈ 1-methylpiperazine > morpholine.

**Figure 5:**
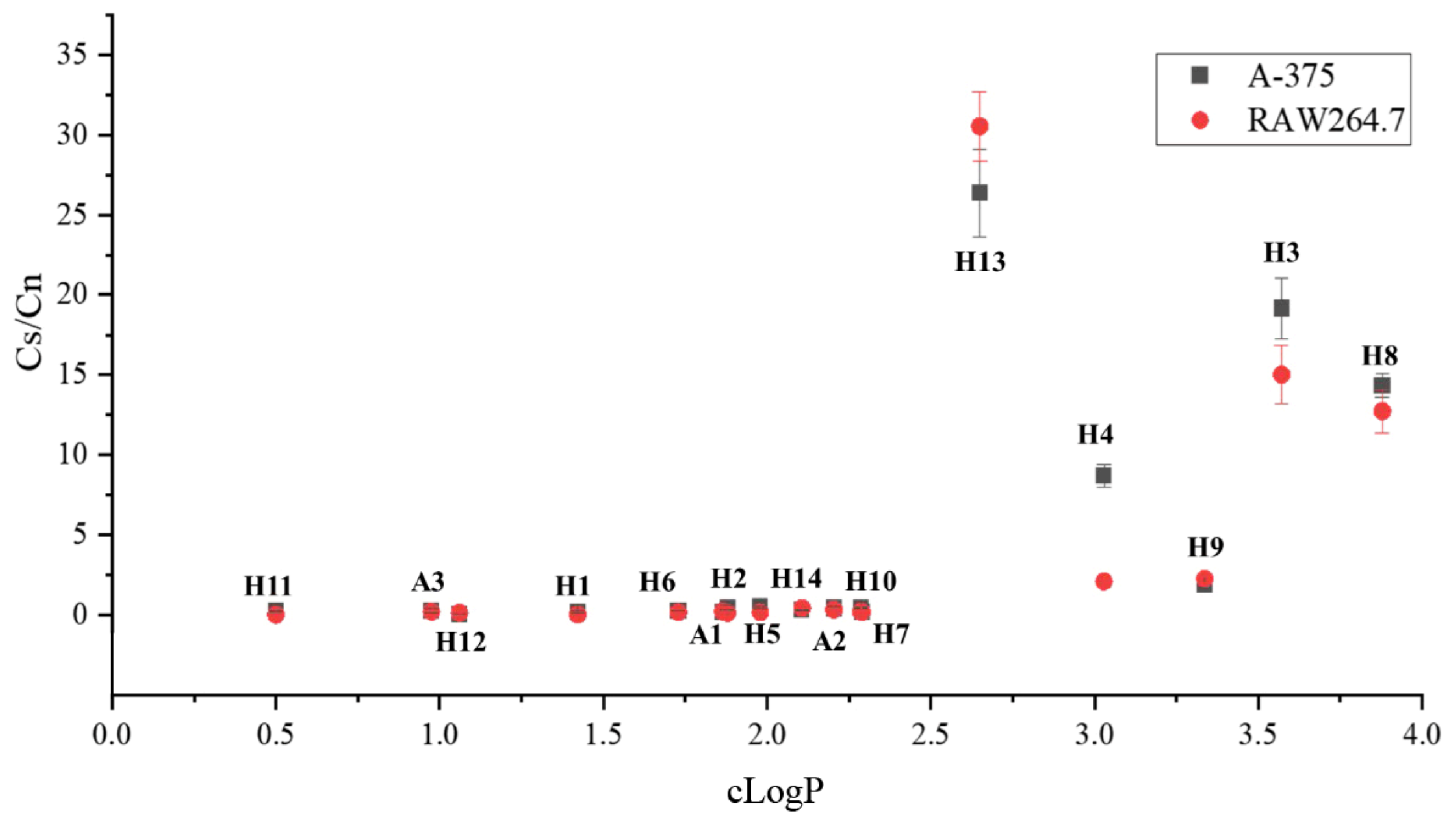
Analysis of the correlation between the cLogP of compounds and cellular uptake. Cs/Cn represents the concentration ratio of the compound to the internal standard NPC in each cell sample (*n* = 3).

Figure 5 illustrates the correlation between cellular uptake efficiency (expressed as the Cs/Cn ratio) and cLogP values of these compounds across two cell lines, A-375 and RAW 264.7. A clear trend emerges from the plot: compounds with a cLogP value below 2.5 generally exhibit low cellular uptake, while those with a cLogP greater than 2.5 exhibit significantly enhanced uptake. Among the latter group, no linear correlation was observed between cellular uptake levels and cLogP values. Compounds **H3**, **H4**, **H8**, and **H13** exhibited particularly high cellular uptake. Their uptake profiles also differed substantially between the two cell lines, implying that the uptake mechanisms may be cell-type dependent. However, this elevated cellular uptake alone does not guarantee cytotoxic activity. Notably, while both **H3** and **H4** exhibited high uptake, they showed no significant cytotoxicity (Figure 1), whereas **H8** and **H13**, which also achieved high intracellular concentrations, displayed potent cytotoxic effects. Collectively, cellular uptake analysis and antiproliferative analysis demonstrated that these compounds with cLogP < 2.5 showed low cellular uptake and minimal antiproliferative activity. Among analogues with cLogP > 2.5, only 5-methoxytryptamine-modified **H8** and **H13** exhibited strong proliferation inhibition, indicating that the 5-methoxytryptamine moiety is essential for their antiproliferative potency. These results indicate that cellular uptake is a necessary prerequisite for these compounds to exert their cytotoxicity, but it is not, in itself, a sufficient condition. Other factors, such as specific intracellular targets, metabolic stability, or the ability to trigger downstream cell death pathways, are likely critical determinants of their ultimate biological activity.

## 3. Materials and Methods

### 3.1. Materials and Reagents

Caffeic acid and LPS were purchased from Shanghai Jizhi Biochemical Technology Co., Ltd. (Shanghai, China). 3,4-Dimethoxycinnamic acid was obtained from Shanghai Accela Technology Co., Ltd. (Shanghai, China). 1-Methylpiperazine and EU were obtained from J&K Scientific Ltd. (Beijing, China). 1-(2-Aminoethyl)piperidine was obtained from Energy Chemical (Shanghai, China). Morpholine was obtained from TCI (Shanghai, China) Development Co., Ltd. (Shanghai, China). 3,4-Methylenedioxycinnamic acid, 2-aminoimidazole sulfate, oxalyl chloride and 5-methoxytryptamine were purchased from Shanghai Aladdin Biochemical Technology Co., Ltd. (Shanghai, China). EdU and the Nitric Oxide Detection Kit were purchased from Beyotime Biotechnology Co., Ltd. (Shanghai, China). DPPH and ABTS were obtained from Merck KGaA (Darmstadt, Germany). All other conventional reagents were purchased from Sinopharm Chemical Reagent Co., Ltd. (Shanghai, China) and Concord Technology Co., Ltd. (Tianjin, China), and used directly without further purification.

### 3.2. Synthesis and Characterization of Caffeic Acid Amide Derivatives

#### 3.2.1. Synthesis of **H1**–**H10**

The acyl chloride method was adopted. At 0 °C, 1.5 mmol of A1 or A2 was added to 30 mL of dichloromethane, and 2 eq of triethylamine was added dropwise and stirred for 5 min; then 0.95 eq of oxalyl chloride was added, and the reaction was carried out for 20–30 min; finally, 1 eq of the amino compound was added, and the reaction was carried out at room temperature for 2–3 h.

#### 3.2.2. Synthesis of **H11**–**H14**

The carbodiimide-type condensing agent method was used. N-Hydroxybenzotriazole (HOBt, 1.2 eq) was added to a solution of caffeic acid (**A3**, 1.5 mmol) in DMF (15 mL), and then 1-(3-dimethylaminopropyl)-3-ethylcarbodiimide (EDCI, 1.2 eq) was added. After stirring for 1 h, a solution of the amino compound (1 eq) in DMF (5 mL) was added, and the solution was stirred at 25 °C for 12 h.

#### 3.2.3. Purification of **H1**–**H14**

The reaction progress was monitored by TLC. After the reaction was completed, 75–120 mL of dichloromethane was added and shaken well, and then 1% HCl solution (100 mL × 2) was added for washing. TLC was used to confirm the phase where the product was located: if it was in the aqueous phase, NaOH solution was added to the aqueous phase to adjust the pH to about 10, and dichloromethane or ethyl acetate was added to extract the product; if it was in the organic phase, saturated NaHCO_3_ solution (50 mL × 3) and pure water (100 mL × 2) were added to wash the organic phase; the organic phase was retained, and an appropriate amount of anhydrous magnesium sulfate was added to remove water overnight. After evaporation of the solvent, the residue was purified by silica gel chromatography to obtain the product.

#### 3.2.4. The Data of NMR and HR-MS of Synthesized Caffeic Acid Amide Derivatives (Figures S3–S44)

^1^H and ^13^C NMR spectra were measured on a Varian/Bruker Avance III HD instrument (equipped with a 400 MHz/54 mm Ascend magnet, operating at 400 MHz and 100 MHz respectively, Bruker BioSpin GmbH, Fällanden, Switzerland) using deuterated dimethyl sulfoxide (DMSO-^d^6) as the solvent and tetramethylsilane (TMS) as the internal standard. Mass spectra were recorded on a Waters Acquity UHPLC system (electrospray ionization, ESI, Waters Corporation, Milford, MA, USA) for ultra-high-performance liquid chromatography–mass spectrometry.

**H1:** 
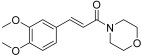

##### (E)-3-(3,4-Dimethoxyphenyl)-1-morpholinoprop-2-en-1-one

Yield (62%). ^1^H NMR (400 MHz, DMSO-*d*_6_) δ 7.46 (d, *J* = 15.3 Hz, 1H), 7.36 (d, *J* = 2.0 Hz, 1H), 7.20 (dd, *J* = 8.3, 2.0 Hz, 1H), 7.10 (d, *J* = 15.2 Hz, 1H), 6.96 (d, *J* = 8.3 Hz, 1H), 3.81 (s, 3H), 3.78 (s, 3H), 3.65–3.51 (m, 8H). ^13^C NMR (101 MHz, DMSO-*d*_6_) δ 165.02, 150.38, 148.99, 142.21, 127.98, 122.56, 115.24, 111.54, 110.31, 66.50, 66.35, 55.74, 55.59, 45.63, 42.18. HR-MS (ESI): *m*/*z* calcd for C_15_H_20_NO_4_ [M + H]^+^: 278.1387; found: 278.1388.

**H2:** 
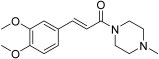

##### (E)-3-(3,4-Dimethoxyphenyl)-1-(4-methylpiperazin-1-yl)prop-2-en-1-one

Yield (55%). ^1^H NMR (400 MHz, DMSO-*d*_6_) δ 7.43 (d, *J* = 15.3 Hz, 1H), 7.36 (d, *J* = 2.0 Hz, 1H), 7.19 (dd, *J* = 8.3, 2.0 Hz, 1H), 7.12 (d, *J* = 15.3 Hz, 1H), 6.95 (d, *J* = 8.3 Hz, 1H), 3.82 (s, 3H), 3.78 (s, 3H), 3.72–3.50 (m, 4H), 2.32 (s, 4H), 2.20 (s, 3H). ^13^C NMR (101 MHz, DMSO-*d*_6_) δ 164.91, 150.36, 149.01, 142.13, 128.03, 122.55, 115.51, 111.55, 110.33, 55.78, 55.61, 55.13, 54.41, 45.52, 44.81, 41.51. HR-MS (ESI): *m*/*z* calcd for C_16_H_23_N_2_O_3_ [M + H]^+^: 291.1703; found: 291.1705.

**H3:** 
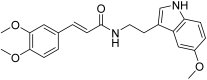

##### (E)-3-(3,4-Dimethoxyphenyl)-N-(2-(5-methoxy-1H-indol-3-yl)ethyl)acrylamide

Yield (75%). ^1^H NMR (400 MHz, DMSO-*d*_6_) δ 10.65 (s, 1H), 8.09 (t, *J* = 5.7 Hz, 1H), 7.35 (d, *J* = 15.7 Hz, 1H), 7.22 (d, *J* = 8.7 Hz, 1H), 7.13 (dd, *J* = 4.3, 2.0 Hz, 2H), 7.09–7.01 (m, 2H), 6.94 (d, *J* = 8.0 Hz, 1H), 6.71 (dd, *J* = 8.7, 2.4 Hz, 1H), 6.47 (d, *J* = 15.7 Hz, 1H), 3.82 (s, 3H), 3.81 (s, 3H), 3.80 (s, 3H), 3.45 (q, *J* = 6.8 Hz, 2H), 2.84 (t, *J* = 7.3 Hz, 2H). ^13^C NMR (101 MHz, DMSO-*d*_6_) δ 165.29, 153.07, 148.50, 148.03, 138.44, 131.47, 129.43, 127.66, 123.44, 123.32, 120.53, 112.12, 111.76, 111.20, 108.68, 106.26, 100.21, 55.50, 55.48, 55.38, 35.90, 25.35. HR-MS (ESI): *m*/*z* calcd for C_22_H_23_N_2_O_4_ [M − H]^−^: 379.1663; found: 379.1665.

**H4:** 
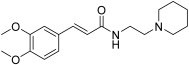

##### (E)-3-(3,4-Dimethoxyphenyl)-N-(2-(piperidin-1-yl)ethyl)acrylamide

Yield (53%). ^1^H NMR (400 MHz, DMSO-*d*_6_) δ 7.91 (s, 1H), 7.34 (d, *J* = 15.7 Hz, 1H), 7.16 (d, *J* = 2.0 Hz, 1H), 7.11 (dd, *J* = 8.3, 2.0 Hz, 1H), 6.97 (d, *J* = 8.3 Hz, 1H), 6.54 (d, *J* = 15.7 Hz, 1H), 3.79 (s, 3H), 3.78 (s, 3H), 3.30 (d, *J* = 19.7 Hz, 2H), 2.51–2.28 (m, 6H), 1.54–1.49 (m, 4H), 1.41–1.35 (m, 2H). ^13^C NMR (101 MHz, DMSO-*d*_6_) δ 165.42, 150.16, 148.96, 138.80, 127.77, 121.51, 119.98, 111.78, 110.00, 57.65, 55.61, 55.50, 53.96, 36.14, 25.25, 23.81. HR-MS (ESI): *m*/*z* calcd for C_18_H_27_N_2_O_3_ [M + H]^+^: 319.2016; found: 319.2015.

**H5:** 
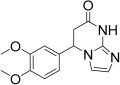

##### (E)-(3,4-Dimethoxyphenyl)-5,6-dihydroimidazo [1,2-a]pyrimidin-7(8H)-one

Yield (52%). ^1^H NMR (400 MHz, DMSO-*d*_6_) δ 11.11 (s, 1H), 6.93 (d, *J* = 8.3 Hz, 1H), 6.87 (d, *J* = 2.1 Hz, 1H), 6.67 (d, *J* = 1.5 Hz, 1H), 6.64 (d, *J* = 1.5 Hz, 1H), 6.52 (dd, *J* = 8.3, 2.1 Hz, 1H), 5.41 (d, *J* = 6.2 Hz, 1H), 3.73 (s, 3H), 3.72 (s, 3H), 3.06 (dd, *J* = 16.3, 6.1 Hz, 1H), 2.93 (dd, *J* = 16.3, 6.3 Hz, 1H). ^13^C NMR (101 MHz, DMSO-*d*_6_) δ 167.22, 149.09, 148.77, 142.54, 131.57, 125.54, 118.20, 114.91, 111.87, 110.23, 55.60, 53.69, 38.74. HR-MS (ESI): *m*/*z* calcd for C_14_H_16_N_3_O_3_ [M + H]^+^: 274.1186; found: 274.1189.

**H6:** 
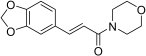

##### (E)-3-(Benzo[d][1,3]dioxol-5-yl)-1-morpholinoprop-2-en-1-one

Yield (62%). ^1^H NMR (400 MHz, DMSO-*d*_6_) δ 7.47 (d, *J* = 1.7 Hz, 1H), 7.43 (d, *J* = 15.3 Hz, 1H), 7.15 (dd, *J* = 8.0, 1.7 Hz, 1H), 7.11 (d, *J* = 15.3 Hz, 1H), 6.93 (d, *J* = 8.0 Hz, 1H), 6.07 (s, 2H), 3.74–3.44 (m, 8H). ^13^C NMR (101 MHz, DMSO-*d*_6_) δ 164.86, 148.65, 148.02, 141.76, 129.66, 124.38, 115.80, 108.43, 106.58, 101.50, 66.49, 66.31, 45.63, 42.18. HR-MS (ESI): *m*/*z* calcd for C_14_H_16_NO_4_ [M + H]^+^: 262.1074; found: 262.1074.

**H7:** 
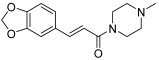

##### (E)-3-(Benzo[d][1,3]dioxol-5-yl)-1-(4-methylpiperazin-1-yl)prop-2-en-1-one

Yield (54%). ^1^H NMR (400 MHz, DMSO-*d*_6_) δ 7.47 (d, *J* = 1.7 Hz, 1H), 7.40 (d, *J* = 15.2 Hz, 1H), 7.14 (d, *J* = 3.0 Hz, 1H), 7.13–7.09 (m, 1H), 6.92 (d, *J* = 8.0 Hz, 1H), 6.06 (s, 2H), 3.68 (s, 2H), 3.54 (s, 2H), 2.31 (d, *J* = 10.1 Hz, 4H), 2.19 (s, 3H) ^13^C NMR (101 MHz, DMSO-*d*_6_) δ 164.72, 148.61, 148.03, 141.62, 129.71, 124.33, 116.09, 108.44, 106.60, 101.49, 55.22, 54.51, 45.68, 44.94, 41.64. HR-MS (ESI): *m*/*z* calcd for C_15_H_19_N_2_O_3_ [M + H]^+^: 275.1390; found: 275.1391.

**H8:** 
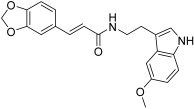

##### (E)-3-(Benzo[d][1,3]dioxol-5-yl)-N-(2-(5-methoxy-1H-indol-3-yl)ethyl)acrylamide

Yield (80%). ^1^H NMR (400 MHz, DMSO-*d*_6_) δ 10.65 (s, 1H), 8.09 (t, *J* = 5.7 Hz, 1H), 7.35 (d, *J* = 15.7 Hz, 1H), 7.22 (d, *J* = 8.7 Hz, 1H), 7.13 (dd, *J* = 4.3, 2.0 Hz, 2H), 7.09–7.01 (m, 2H), 6.94 (d, *J* = 8.0 Hz, 1H), 6.71 (dd, *J* = 8.7, 2.4 Hz, 1H), 6.47 (d, *J* = 15.7 Hz, 1H), 6.06 (s, 2H), 3.75 (s, 3H), 3.45 (q, *J* = 6.8 Hz, 2H), 2.84 (t, *J* = 7.3 Hz, 2H). ^13^C NMR (101 MHz, DMSO-*d*_6_) δ 165.29, 153.07, 148.50, 148.03, 138.44, 131.47, 129.43, 127.66, 123.44, 123.32, 120.53, 112.12, 111.76, 111.20, 108.68, 106.26, 101.52, 100.21, 55.38, 35.90, 25.35. HR-MS (ESI): *m*/*z* calcd for C_21_H_21_N_2_O_4_ [M + H]^+^: 365.1496; found: 365.1497.

**H9:** 
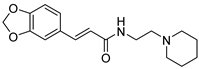

##### (E)-3-(Benzo[d][1,3]dioxol-5-yl)-N-(2-(piperidin-1-yl)ethyl)acrylamide

Yield (56%). ^1^H NMR (400 MHz, DMSO-*d*_6_) δ 7.87 (t, *J* = 5.7 Hz, 1H), 7.31 (d, *J* = 15.7 Hz, 1H), 7.14 (d, *J* = 1.7 Hz, 1H), 7.05 (dd, *J* = 8.0, 1.7 Hz, 1H), 6.94 (d, *J* = 8.0 Hz, 1H), 6.50 (d, *J* = 15.7 Hz, 1H), 6.06 (s, 2H), 3.26 (q, *J* = 6.5 Hz, 2H), 2.33 (d, *J* = 6.8 Hz, 6H), 1.49 (q, *J* = 5.5 Hz, 4H), 1.37 (q, *J* = 5.6 Hz, 2H). ^13^C NMR (101 MHz, DMSO-*d*_6_) δ 165.21, 148.48, 148.00, 138.41, 129.40, 123.35, 120.44, 108.63, 106.23, 101.50, 57.83, 54.10, 36.36, 25.50, 24.05. HR-MS (ESI): *m*/*z* calcd for C_17_H_23_N_2_O_3_ [M + H]^+^: 303.1703; found: 303.1703.

**H10:** 
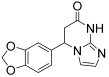

##### 5. -(Benzo[d][1,3]dioxol-5-yl)-5,6-dihydroimidazo [1,2-a]pyrimidin-7(8H)-one

Yield (52%). ^1^H NMR (400 MHz, DMSO-*d*_6_) δ 11.14 (s, 1H), 6.90 (d, *J* = 8.0 Hz, 1H), 6.76 (d, *J* = 1.9 Hz, 1H), 6.69–6.64 (m, 2H), 6.54 (dd, *J* = 7.9, 1.9 Hz, 1H), 6.02 (s, 2H), 5.42 (d, *J* = 6.1 Hz, 1H), 3.07 (dd, *J* = 16.2, 6.1 Hz, 1H), 2.89 (d, *J* = 4.8 Hz, 1H). ^13^C NMR (101 MHz, DMSO-*d*_6_) δ 167.12, 147.90, 147.27, 142.49, 133.17, 125.64, 119.70, 114.92, 108.50, 106.67, 101.40, 53.64, 38.72. HR-MS (ESI): *m*/*z* calcd for C_13_H_12_N_3_O_3_ [M + H]^+^: 258.0873; found: 258.0874.

**H11:** 
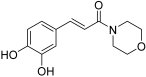

##### (E)-3-(3,4-Dihydroxyphenyl)-1-morpholinoprop-2-en-1-one

Yield (61%). ^1^H NMR (400 MHz, DMSO-*d*_6_) δ 9.44 (s, 1H), 8.96 (s, 1H), 7.34 (d, *J* = 15.3 Hz, 1H), 7.08 (d, *J* = 2.1 Hz, 1H), 6.98 (dd, *J* = 8.2, 2.1 Hz, 1H), 6.91 (d, *J* = 15.3 Hz, 1H), 6.74 (d, *J* = 8.1 Hz, 1H), 3.77–3.39 (m, 8H). ^13^C NMR (101 MHz, DMSO-*d*_6_) δ 165.10, 147.50, 145.50, 142.50, 126.75, 120.79, 115.65, 114.97, 114.02, 66.40, 65.66, 45.61, 42.12. HR-MS (ESI): *m*/*z* calcd for C_13_H_14_NO_4_ [M − H]^−^: 248.0928; found: 248.0929.

**H12:** 
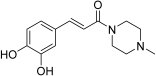

##### (E)-3-(3,4-Dihydroxyphenyl)-1-(4-methylpiperazin-1-yl)prop-2

Yield (22%). ^1^H NMR (400 MHz, DMSO-*d*_6_) δ 9.44 (s, 1H), 8.96 (s, 1H), 7.31 (d, *J* = 15.2 Hz, 1H), 7.08 (d, *J* = 2.1 Hz, 1H), 6.98 (dd, *J* = 8.2, 2.1 Hz, 1H), 6.92 (d, *J* = 15.2 Hz, 1H), 6.74 (d, *J* = 8.1 Hz, 1H), 3.65 (s, 2H), 3.55 (s, 2H), 2.33 (s, 4H), 2.21 (s, 3H). ^13^C NMR (101 MHz, DMSO-*d*_6_) δ 164.98, 147.49, 145.53, 142.42, 126.79, 120.77, 115.70, 114.97, 114.25, 55.10, 54.35, 45.54, 44.81, 41.47. HR-MS (ESI): *m*/*z* calcd for C_14_H_17_N_2_O_3_ [M − H]^−^: 261.1245; found: 261.1246.

**H13:** 
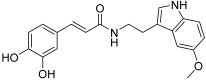

##### (E)-3-(3,4-Dihydroxyphenyl)-N-(2-(5-methoxy-1H-indol-3-yl)ethyl)acrylamide

Yield (30%). ^1^H NMR (400 MHz, DMSO-*d*_6_) δ 10.64 (s, 1H), 9.34 (s, 1H), 9.12 (s, 1H), 8.09 (t, *J* = 5.8 Hz, 1H), 7.28–7.19 (m, 2H), 7.12 (d, *J* = 2.3 Hz, 1H), 7.03 (d, *J* = 2.5 Hz, 1H), 6.94 (d, *J* = 2.0 Hz, 1H), 6.83 (dd, *J* = 8.2, 2.1 Hz, 1H), 6.74 (d, *J* = 8.1 Hz, 1H), 6.71 (dd, *J* = 8.7, 2.5 Hz, 1H), 6.34 (d, *J* = 15.6 Hz, 1H), 3.74 (s, 3H), 3.43 (q, *J* = 6.9 Hz, 2H), 2.84 (t, *J* = 7.3 Hz, 2H). ^13^C NMR (101 MHz, DMSO-*d*_6_) δ 165.58, 153.07, 147.35, 145.62, 139.12, 131.47, 127.69, 126.53, 123.44, 120.50, 118.75, 115.86, 113.91, 112.13, 111.84, 111.22, 100.21, 55.39, 48.72, 25.39. HR-MS (ESI): *m*/*z* calcd for C_20_H_19_N_2_O_4_ [M − H]^−^: 351.1350; found: 351.1352.

**H14:** 
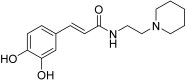

##### (E)-3-(3,4-Dihydroxyphenyl)-N-(2-(piperidin-1-yl)ethyl)acrylamide

Yield (23%). ^1^H NMR (400 MHz, DMSO-*d*_6_) δ 9.40 (s, 1H), 9.14 (s, 1H), 7.87 (t, *J* = 5.7 Hz, 1H), 7.31 (d, *J* = 15.7 Hz, 1H), 7.14 (d, *J* = 1.7 Hz, 1H), 7.05 (dd, *J* = 8.0, 1.7 Hz, 1H), 6.94 (d, *J* = 8.0 Hz, 1H), 6.50 (d, *J* = 15.7 Hz, 1H), 3.26 (q, *J* = 6.5 Hz, 2H), 2.33 (d, *J* = 6.8 Hz, 6H), 1.49 (q, *J* = 5.5 Hz, 4H), 1.37 (q, *J* = 5.6 Hz, 2H). ^13^C NMR (101 MHz, DMSO-*d*_6_) δ 165.97, 147.58, 145.68, 139.70, 126.28, 120.66, 118.12, 115.91, 113.99, 56.30, 52.92, 48.70, 23.47, 22.26. HR-MS (ESI): *m*/*z* calcd for C_16_H_23_N_2_O_3_ [M + H]^+^: 291.1703; found: 291.1703.

### 3.3. Cell Lines and Cell Culture

Cell lines: HeLa (human cervical cancer cells), DU145 (human prostate cancer cells), A549 (human non-small cell lung cancer cells), K-562 (human chronic myeloid leukemia cells), LoVo (human colon cancer cells), MNK-45 (human gastric cancer cells), U-87 MG (human glioblastoma astrocytoma cells), RAW264.7 (mouse monocyte macrophage leukemia cells), SH-SY5Y (human neuroblastoma cells), and L-02 (human normal hepatocyte cells) were purchased from the Cell Resource Center of the Institute of Basic Medical Sciences, Chinese Academy of Medical Sciences (Beijing, China); A431 (human epidermal cancer cells) were kindly provided by the group of Wang Fuyi from the Institute of Chemistry, Chinese Academy of Sciences (Beijing, China); A-375 (human malignant melanoma cells) were kindly provided by Beijing Cancer Hospital (Beijing, China); A2780T (paclitaxel-resistant human ovarian cancer cell line) was purchased from Jiangsu KeyGen Biotech Co., Ltd. (Nanjing, China); THP-1 (human monocytic leukemia cells), BeWo (human choriocarcinoma cells) were purchased from the China Center for Type Culture Collection at Wuhan University (Wuhan, China); EBC-1 (human lung squamous cell carcinoma cells) was purchased from Bohui Biotechnology Co., Ltd. (Guangzhou, China); LX-2 (human hepatic stellate cells) was purchased from Beijing Purosin Technology Co., Ltd. (Beijing, China).

Cell culture: Unless otherwise specified, all cells were routinely cultured in a passage incubator (37 °C, 5% CO_2_). The preparation of the complete medium is as follows: supplement the medium with 10% fetal bovine serum (FBS, Gibco, Waltham, MA, USA) and 1% penicillin/streptomycin (Corning, Corning, NY, USA). Except that U-87 MG, A431, A-375 and RAW264.7 cells use DMEM medium (Gibco), and SH-SY5Y cells use RPMI-1640 medium (Gibco) with 15% FBS, the rest of the cells use RPMI-1640 medium.

### 3.4. Cell Proliferation Assay

#### 3.4.1. 120 h Cell Proliferation Assay

HeLa, DU145, A549, A431, A-375, U87-MG, and A2780T cells in the logarithmic growth phase were seeded into 96-well plates at a density of 3000 cells/well and pre-cultured for 12 h. Subsequently, gradient dilutions of 14 caffeic acid derivatives, along with 3,4-dimethoxycinnamic acid (**A1**), 3,4-methylenedioxycinnamic acid (**A2**), caffeic acid (**A3**), and caffeic acid phenethyl ester (**CAPE**), were added to parallel wells (final concentrations set at 0, 2, 10, and 50 μM). After 72 h of culture, the medium was removed and replaced with 200 μL/well of fresh medium containing the same concentration of compounds, followed by continued culture for 48 h.

#### 3.4.2. 48 h Cell Proliferation Assay

HeLa, A431, A-375, THP-1, MKN-45, A2780T, BeWo, BC-1, LX-2, K562, LoVo, RAW264.7, and L-02 cells in the logarithmic growth phase were seeded into 96-well plates at a density of 5000 cells/well and pre-cultured for 12 h. Gradient dilutions of H8, H13, and CAPE were added to parallel wells (final concentrations set at 0, 0.195, 0.781, 3.12, 12.5, 50, and 200 μM) and cultured for 48 h.

Following incubation, the medium was removed from each well, and RPMI-1640 medium containing 10% CCK-8 was added. Plates were incubated for an additional 0.5–1 h. All samples were run in triplicate, and absorbance at 450 nm was measured using a SpectraMax M2 microplate reader (Molecular Devices, LLC, San Jose, CA, USA). Relative cell viability was calculated using the formula: Relative cell viability (%) = [(A_S_ − A_B_)/(A_C_ − A_B_)] × 100%, where A_S_ is the absorbance of the compound-treated group, A_C_ is the absorbance of the untreated control group, and A_B_ is the absorbance of the blank group containing only CCK-8 and medium.

### 3.5. The De Novo Synthesis of Cellular DNA and RNA

A-375 cells in the logarithmic growth phase were seeded into 6-well plates at a density of 5 × 10^5^ cells/well. After 12 h of pre-culture, **H8** and **H13** were added to each well at final concentrations of 10 μM or 30 μM, respectively, followed by continuous culture for 24 h. Subsequently, according to the instructions of the BeyoClick™ EdU Cell Proliferation Kit with Alexa Fluor 647 (Beyotime Biotech Inc., Shanghai, China) and literature recommendations, 10 μM EdU or 1 mM EU was added to each well, and the cells were incubated for 3.5 h for labeling. After labeling, the cells were washed twice with PBS containing 3% BSA, then harvested by trypsin digestion. The cells were fixed with 4% paraformaldehyde at 4 °C for 30 min, permeabilized with 0.3% Triton X-100 for 20 min, and then incubated with Click reaction solution (containing Azide 647, CuSO_4_, Click Reaction Buffer, and Click Additive Solution) for 30 min at room temperature in the dark. After the reaction, the cells were washed once with PBS. The newly synthesized DNA or RNA was detected using a Becton Dickinson FACScalibur flow cytometer (BD Biosciences, Franklin Lakes, NJ, USA).

### 3.6. Cell Cycle Analysis

A-375 cells in the logarithmic growth phase were seeded into 6-well plates at a density of 5 × 10^5^ cells/well. After 12 h of pre-culture, **H8** and **H13** were added to each well at final concentrations of 10 μM or 30 μM, respectively, followed by another 24 h of culture. Cells in the supernatant were collected, and the remaining cells were digested with EDTA-free trypsin and harvested. After washing with PBS, the cells were fixed with 75% ethanol at -20 °C for 12 h. After removing ethanol, the cell cycle distribution was evaluated using a DNA Content Quantitation Assay Kit: 50 μL of RNase A solution was added first and incubated at 37 °C for 30 min, then 200 μL of Propidium Iodide (PI) staining solution was added and incubated at 4 °C in the dark for 30 min. DNA content was detected using a Becton Dickinson FACSCalibur flow cytometer.

### 3.7. Antioxidant Capacity Testing

#### 3.7.1. Analysis of DPPH Radical Scavenging Rate

In a 96-well plate, 80 μL of 0.2 mM DPPH radical ethanol solution was added to an equal volume of ethanol solutions of compounds at different concentrations (final concentrations were 1.37, 4.11, 12.35, 37.04, 111.11, 333.33 and 1000 μM). After incubation in the dark for 30 min, the absorbance at 517 nm was measured using a SpectraMax M2 microplate reader.

#### 3.7.2. Analysis of ABTS Radical Scavenging Rate

Dissolve ABTS in a sodium acetate solution with a pH of 4.5 to prepare a 7.4 mM ABTS solution. Mix the ABTS solution with a 2.6 mM potassium persulfate solution at a volume ratio of 1:1, let it stand in the dark at room temperature for 16 h, and then dilute the prepared ABTS solution with ethanol to an absorbance of around 0.7 at 723 nm. In a 96-well plate, add 160 μL of the ABTS free radical ethanol solution to 40 μL of ethanol solutions of different concentrations of the compound (final concentrations are 7.8125, 15.625, 31.25, 62.5, 125, 250, and 500 μM). After shaking in the dark for 4 min, measure the absorbance at 723 nm using a SpectraMax M2 microplate reader.

Three replicate wells were set for all test samples. The free radical scavenging rate (S%) is calculated as follows: S(%) = (A_b_ − A_s_)/A_b_ × 100%, where A_b_ is the absorbance of the blank sample and A_s_ is the absorbance of the tested sample.

### 3.8. Detection of Nitric Oxide Content

RAW264.7 cells in the logarithmic growth phase were seeded in 12-well plates at a density of 2 × 10^5^ cells per well. After pre-culturing for 12 h, compounds with a final concentration of 100 μM were added and incubated for 1 h, and then lipopolysaccharide (LPS) with a final concentration of 1 µg/mL was added and cultured for another 48 h. According to the experimental protocol of the Nitric Oxide Detection Kit, in a 96-well plate, 50 μL of Griess Reagent I was first added, then 50 μL of the supernatant of the cell culture medium was added, and finally 50 μL of Griess Reagent II was added. The plate was incubated for 5 min with shaking in the dark. The absorbance at 525 nm was measured using a SpectraMax M2 microplate reader. Three replicate wells were set for all test samples.

### 3.9. Antibacterial Activity Study

The minimum inhibitory concentration (MIC) of the synthesized caffeic acid amide derivatives was determined by the broth microdilution method. The detailed procedures are described as follows. Test strains were inoculated into Mueller-Hinton Broth (MHB) and incubated with shaking until the OD_600_ value reached 1.0. The bacterial culture was subcultured at an inoculum size of 1% (*v*/*v*) in fresh MHB and further incubated until OD_600_ = 1.0, corresponding to a bacterial concentration of 1.5 × 10^8^ CFU/mL. The culture was first diluted 10-fold with sterile water to obtain a suspension with OD_600_ = 0.1, and then diluted 300-fold with MHB to a final concentration of 5 × 10^5^ CFU/mL. The diluted bacterial suspension was added to sterile 96-well plates, followed by the addition of different caffeic acid amide derivatives at various concentrations and the positive control drug levofloxacin. After incubation at 37 °C for 15 h, the experimental results were observed and recorded.

### 3.10. Neuroprotective Study

#### 3.10.1. Establishment of Alzheimer’s Disease Cell Model

According to the suggestion in the article, SH-SY5Y cells in the logarithmic growth phase were seeded in 96-well plates at a density of 5000 cells/well and pre-cultured for 12 h. Then, different concentrations of H_2_O_2_ were added and incubated for 48 h (final concentrations were 0.08, 0.31, 1.22, 4.88, 19.53, 78.13, 312.5, 1250, and 5000 μM). The culture medium was removed, and RPMI-1640 medium containing 10% CCK-8 was added, followed by continued incubation for 0.5–1 h. Three replicate wells were set for all samples. The absorbance at 450 nm was measured using a SpectraMax M2 microplate reader.

#### 3.10.2. Evaluation of Neuroprotective Effect

SH-SY5Y cells in the logarithmic growth phase were seeded in 96-well plates at a density of 5000 cells/well and pre-cultured for 12 h. Compounds at different concentrations (2, 10, and 50 μM) were added and incubated for 2 h, and then H_2_O_2_ at a final concentration of 300 μM was added and incubated for 48 h. The culture medium was removed, and RPMI-1640 medium containing 10% CCK-8 was added, followed by continued incubation for 0.5–1 h. Three replicate wells were set for all samples. The absorbance at 450 nm was measured using a SpectraMax M2 microplate reader, and the relative cell viability was calculated.

### 3.11. LC—MS Analysis of the Cellular Uptake of Compounds

#### 3.11.1. Sample Preparation

Seed A-375 and RAW264.7 cells separately into 12-well plates at a density of 1 × 10^6^ cells per well. After 24 h, add **H1**–**H14**, and **A1**–**A3** respectively and NPC (each 100 μM; NPC serves as the internal reference). Following 1 h of co-incubation, wash the cells five times with PBS, then harvest the cells. Add acetonitrile to lyse the cells by ultrasonication, centrifuge the mixture and collect the supernatant for subsequent LC-MS analysis.

#### 3.11.2. Chromatography

Samples were loaded using a Waters Acquity UPLC Class I system equipped with an autosampler (Waters Corporation, Milford, MA, USA). A Waters BEH C18 reversed-phase column (1.7 μm, 2.1 mm × 150 mm) was used, and the column temperature was maintained at 40 °C. Mobile phase A was water containing 0.5% formic acid, and mobile phase B was acetonitrile-water (8:2, *v*/*v*) containing 0.5% formic acid. The total run time for each sample was 10 min with the following gradient elution program: (1) 0.0–1.0 min, 35% B; (2) 1.0–4.0 min, 35% B to 65% B; (3) 4.0–8.0 min, 65% B; (4) 8.0–9.0 min, 65% B to 35% B; (5) 9.0–10.0 min, 35% B. The flow rate was set at 300 μL/min.

#### 3.11.3. Mass Spectrometry

Mass spectrometry analysis was performed using a SCIEX 4500 QTRAP mass spectrometer equipped with an electrospray ionization (ESI) source (SCIEX, Framingham, MA, USA). Nitrogen was used as both the nebulizer gas and desolvation gas. The typical operating parameters were set as follows: curtain gas (CUR) at 25, collision gas (CAD) set to medium, ion source temperature at 550 °C, ion source gas 1 (GS1) at 45, ion source gas 2 (GS2) at 50, and electrospray voltage at 4500 V. The detection mode was positive ion mode. The precursor ions (Q1) and corresponding declustering potential (DP) of each compound were acquired. Under the optimized DP, the characteristic fragment ions (Q3) and corresponding collision energy (CE) for each compound were determined.

## 4. Conclusions

In summary, fourteen caffeic acid amide derivatives (**H1**–**H14**) were rationally designed and synthesized through amidation reactions, followed by comprehensive biological evaluation. Compounds with a clogP value greater than 2.5 exhibited enhanced cellular uptake. **H8** and **H13** incorporating 5-methoxytryptamine, strongly suppressed tumor cell proliferation by inhibiting DNA synthesis and arresting the cell cycle at the G_2_/M phase. Compounds bearing a catechol group, namely **H11**, **H12**, **H13** and **H14**, possessed favorable antioxidant activity. All synthesized compounds inhibited LPS-stimulated NO production. Among these, **H11** and **H12** exhibited the most potent activity with no detectable cytotoxicity, demonstrating strong anti-inflammatory effects. Additionally, **H3**, **H4**, **H5**, **H6**, **H7**, **H11** and **H12** markedly alleviated H_2_O_2_-induced neuronal damage, and **H5** exhibited the strongest protective activity. This work broadens the structural diversity of caffeic acid derivatives and illustrates the regulatory effects of 3,4-substitutions and carboxyl amidation on bioactivities. It offers new guidance for developing high-potency caffeic acid derivatives.

## Figures and Tables

**Table 1:** The IC_50_ values of CAPE, H8, and H13 for different cells (*n* = 3).

	IC_50_ (μM)		
Cell Line	CAPE	H8	H13
HeLa	27.26 ± 0.74	9.56 ± 0.78	25.26 ± 1.42
A431	32.41 ± 1.22	15.53 ± 0.46	61.70 ± 3.39
A-375	24.28 ± 1.35	7.65 ± 0.49	20.28 ± 4.31
THP-1	51.24 ± 9.62	32.67 ± 2.46	76.09 ± 13.9
MKN-45	>100	25.58 ± 1.02	25.58 ± 4.03
A2780T	38.25 ± 2.48	39.06 ± 3.19	>100
BeWo	14.70 ± 4.16	17.32 ± 2.22	91.19 ± 8.89
EBC-1	>100	21.41 ± 1.55	6.75 ± 0.30
LX-2	45.48 ± 3.97	40.14 ± 2.12	63.71 ± 6.98
K562	25.77 ± 2.87	4.99 ± 0.39	17.32 ± 3.31
LoVo	50.28 ± 6.53	11.57 ± 1.10	56.61 ± 2.57
RAW264.7	1.11 ± 0.11	47.54 ± 3.85	10.93 ± 0.79
L-02	76.41 ± 1.09	10.67 ± 1.11	75.69 ± 1.10

**Table 2:** Antioxidant activity in vitro of the tested compounds (*n* = 3).

EC_50_ (μM)		
Compounds	DPPH	ABTS
Caffeic acid (A3)	19.13 ± 1.06	48.02 ± 1.90
CAPE	15.15 ± 0.40	49.77 ± 2.68
H11	14.58 ± 0.56	49.79 ± 6.21
H12	35.80 ± 1.70	93.20 ± 7.41
H13	19.89 ± 0.70	44.79 ± 2.63
H14	98.95 ± 5.41	180.53 ± 8.28

## Data Availability

Data is contained within the article or Supplementary Materials.

## Supplementary Materials

The following supporting information can be downloaded at: https://www.mdpi.com/article/10.3390/ijms27188262/s1.
