# Supplementary material for: Synthesis and Biological Activity Assessment of Caffeic Acid Azaheterocyclic Amide Derivatives
Source: Int J Mol Sci. 2026 Sep 16;27(18):8262. doi: 10.3390/ijms27188262 (PMC13607599; doi:10.3390/ijms27188262)
Supplement: Supplementary file 1 [file ijms-27-08262-s001.zip › ijms-4504078-supplementary.pdf]

**Table S1.** MIC of the compounds (μg/mL) on different bacteria.

| Compounds | Gram-negative bacteria | | | Gram-positive bacteria | | |
|---|---|---|---|---|---|---|
| | Escherichia coli | Pseudomonas aeruginosa | Enterococcus faecium | Enterococcus faecium | Enterococcus faecium | Enterococcus faecium (resistant to vancomycin) |
| H1 | >256 | >256 | >256 | >256 | >256 | >256 |
| H2 | >256 | >256 | >256 | >256 | >256 | >256 |
| H3 | >256 | >256 | >256 | >256 | >256 | >256 |
| H4 | >256 | >256 | >256 | >256 | >256 | >256 |
| H5 | >256 | >256 | >256 | >256 | >256 | >256 |
| H6 | >256 | >256 | >256 | >256 | >256 | >256 |
| H7 | >256 | >256 | >256 | >256 | >256 | >256 |
| H8 | >256 | >256 | >256 | >256 | >256 | >256 |
| H9 | >256 | >256 | >256 | >256 | >256 | >256 |
| H10 | >256 | >256 | >256 | >256 | >256 | >256 |
| H11 | >256 | >256 | >256 | >256 | >256 | >256 |
| H12 | >256 | >256 | >256 | >256 | >256 | >256 |
| H13 | 256 | 256 | 128 | 256 | >256 | 256 |
| H14 | >256 | >256 | >256 | >256 | >256 | >256 |
| 5-MT | >256 | >256 | >256 | >256 | >256 | >256 |
| CAPE | 128 | >256 | 32 | 64 | 128 | 64 |
| A1 | >256 | >256 | >256 | >256 | >256 | >256 |
| A2 | >256 | >256 | >256 | >256 | >256 | >256 |
| A3 | >256 | >256 | >256 | >256 | >256 | >256 |
| levofloxacin | ≤0.5 | ≤0.5 | ≤0.5 | ≤0.5 | ≤0.5 | 32 |

**Table S2.** The cLogP values of the compounds.

| Compounds | cLogP | Compounds | cLogP |
|---|---|---|---|
| H1 | 1.422 | H10 | 2.286 |
| H2 | 1.864 | H11 | 0.500 |
| H3 | 3.571 | H12 | 1.061 |
| H4 | 3.029 | H13 | 2.649 |
| H5 | 1.979 | H14 | 2.107 |
| H6 | 1.729 | A1 | 1.879 |
| H7 | 2.290 | A2 | 2.204 |
| H8 | 3.878 | A3 | 0.975 |
| H9 | 3.337 | | |

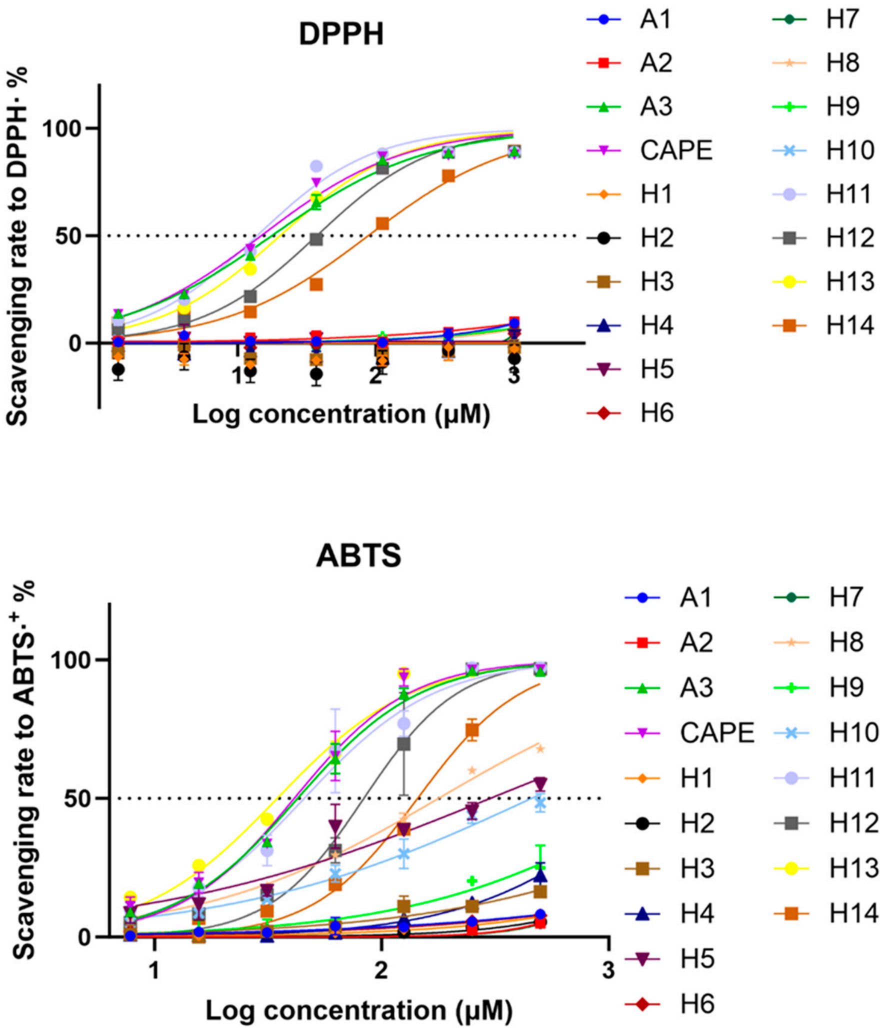


**Figure S1**. The DPPH and ABTS radical scavenging abilities of the compounds.

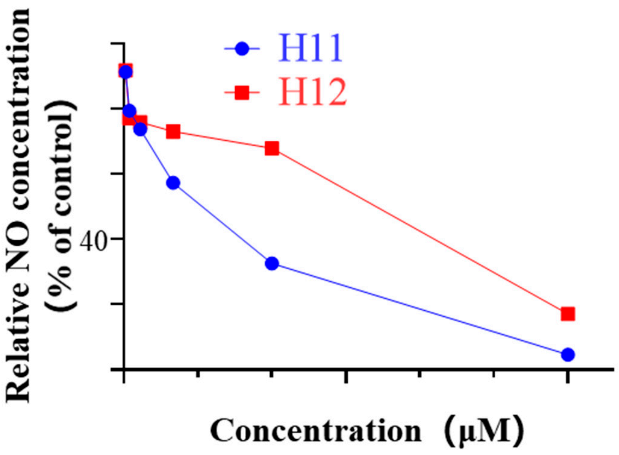


**Figure S2.** The anti-inflammatory response curves of H11 and H12 in RAW264.7 macrophages.

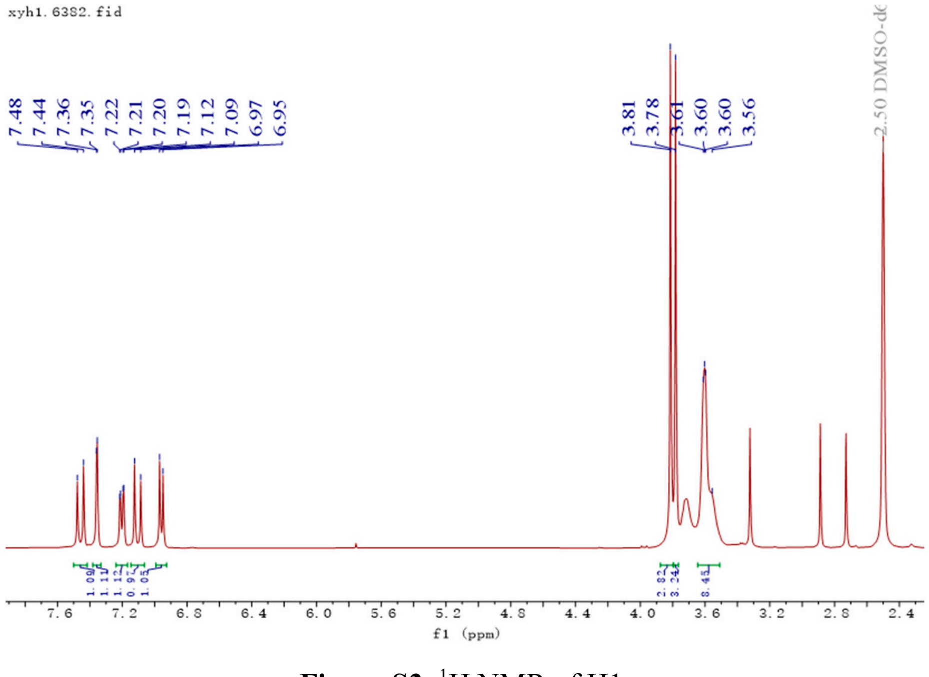


**Figure S3.** $^{1}$H NMR of H1.

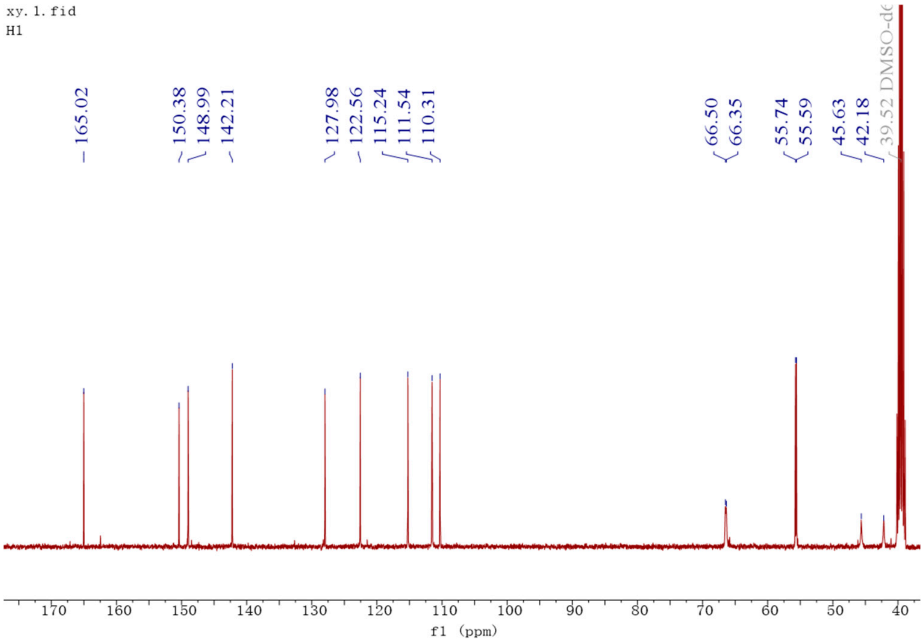


**Figure S4.** $^{13}$C NMR of H1.

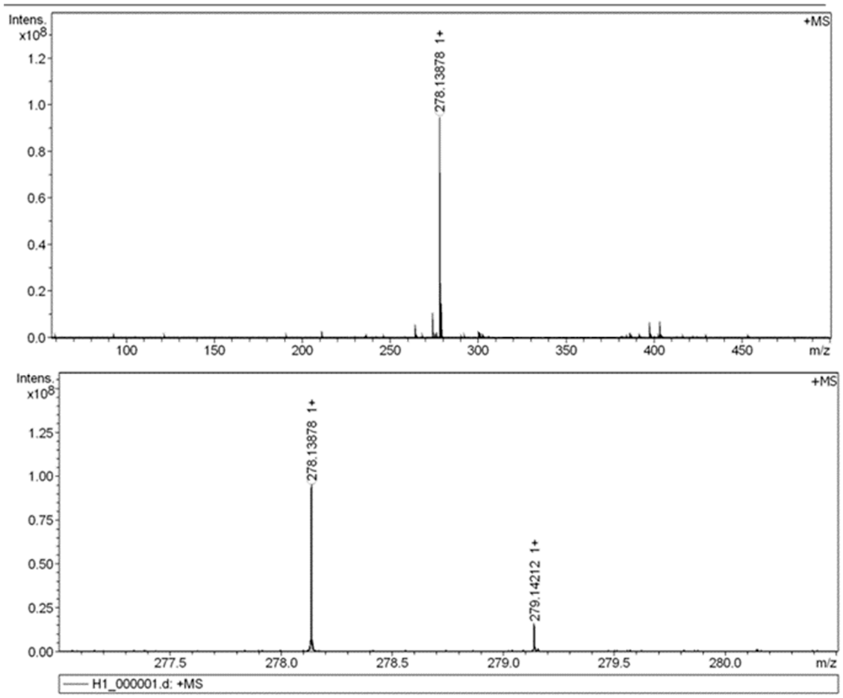


| Meas. m/z | # | Ion Formula | Score | m/z | err [ppm] | Mean err [ppm] | mSigma | rdb | e⁻ Conf | N-Rule |
|---|---|---|---|---|---|---|---|---|---|---|
| 278.138778 | 1 | C15H20NO4 | 100.00 | 278.138685 | -0.3 | -0.3 | 10.4 | 6.5 | even | ok |

**Figure S5.** HR-MS (ESI) of H1.

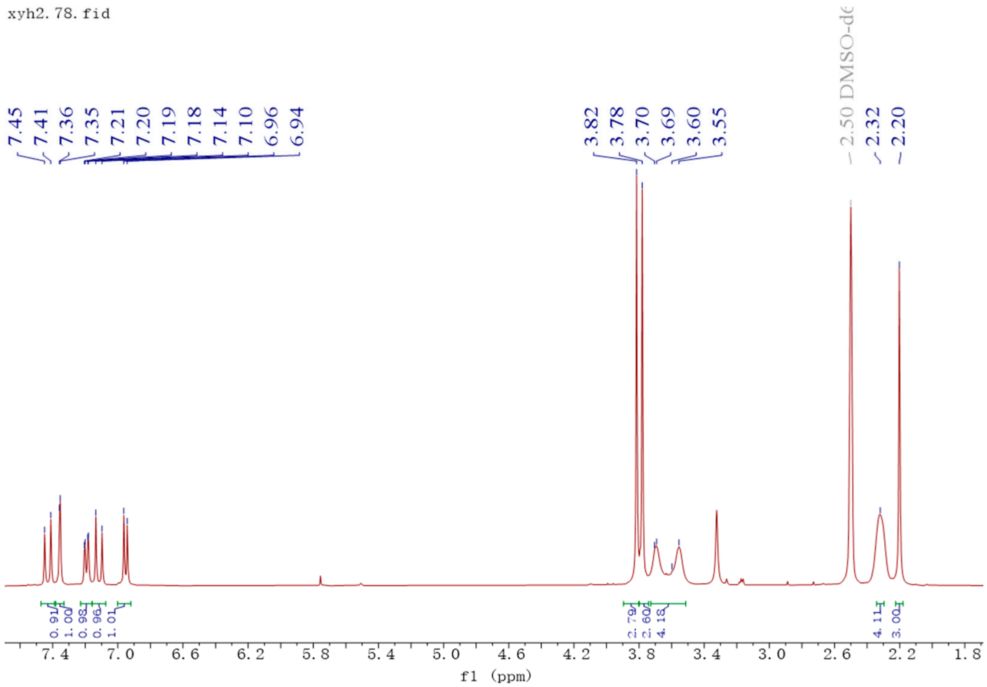


**Figure S6.** $^{1}$H NMR of H2.

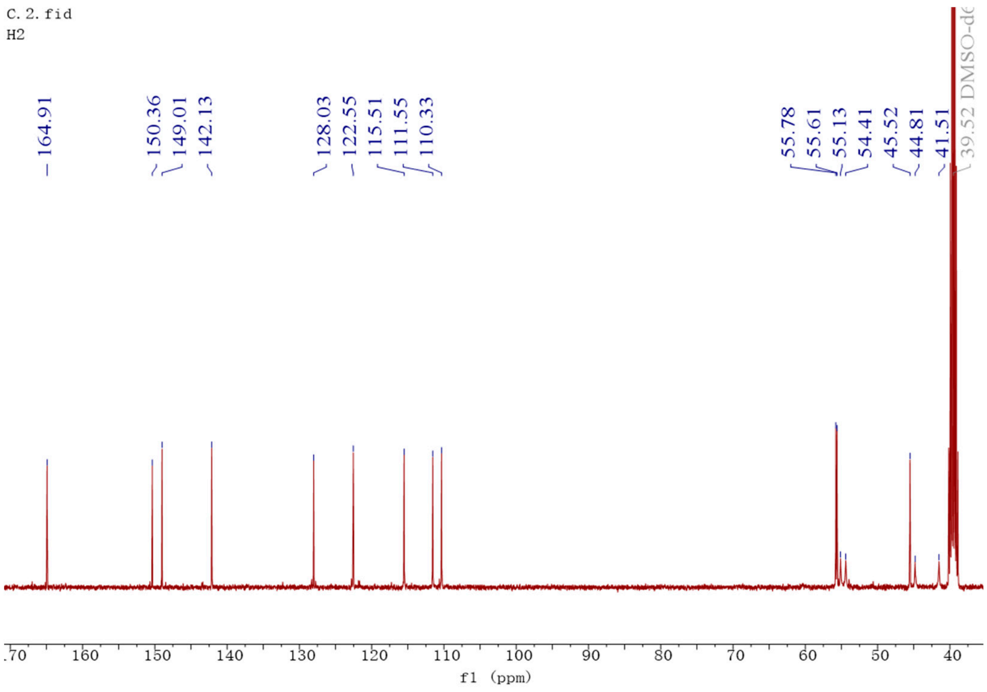


**Figure S7.** $^{13}$C NMR of H2.

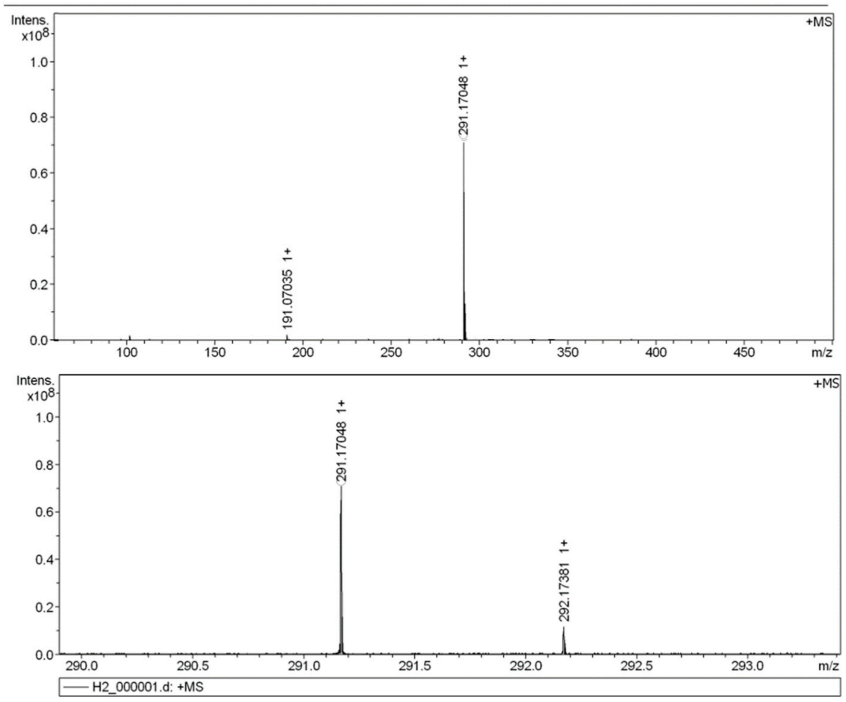


| Meas. m/z | # | Ion Formula | Score | m/z | err [ppm] | Mean err [ppm] | mSigma | rdb | e⁻ Conf | N-Rule |
|---|---|---|---|---|---|---|---|---|---|---|
| 291.170482 | 1 | C16H23N2O3 | 100.00 | 291.170319 | -0.6 | -0.7 | 10.3 | 6.5 | even | ok |

**Figure S8.** HR-MS (ESI) of H2.

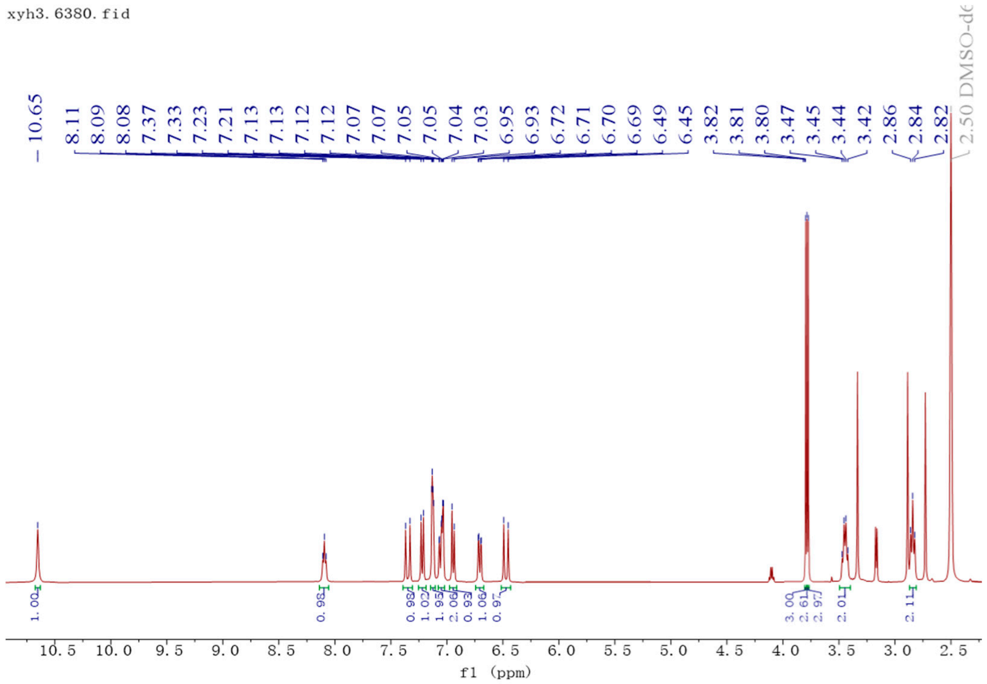


**Figure S9.** $^{1}$H NMR of H3.

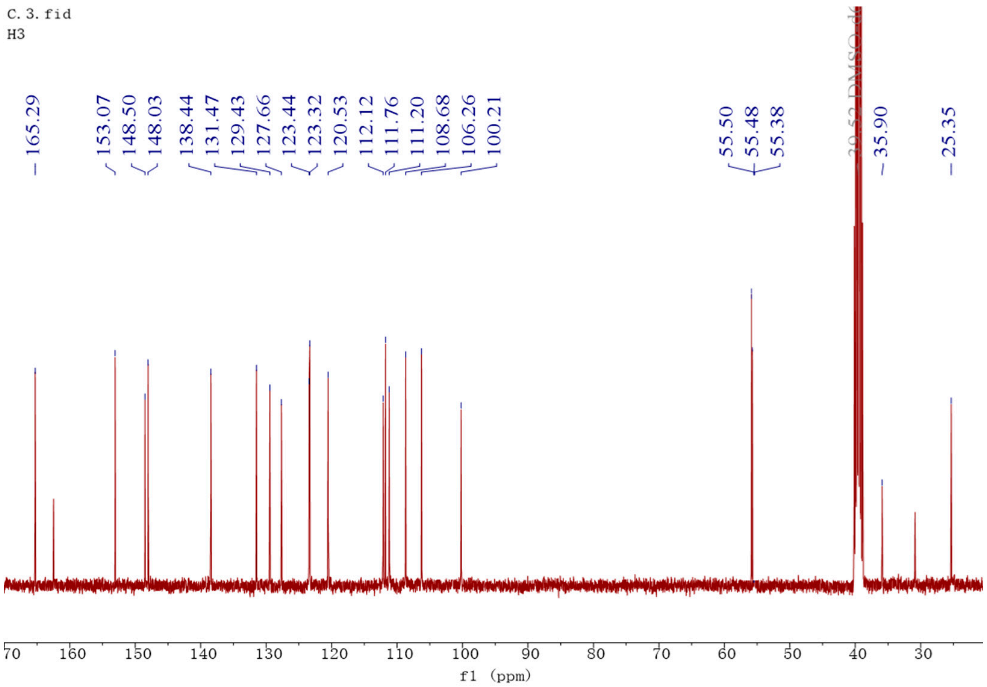


**Figure S10.** $^{13}C$ NMR of H3.

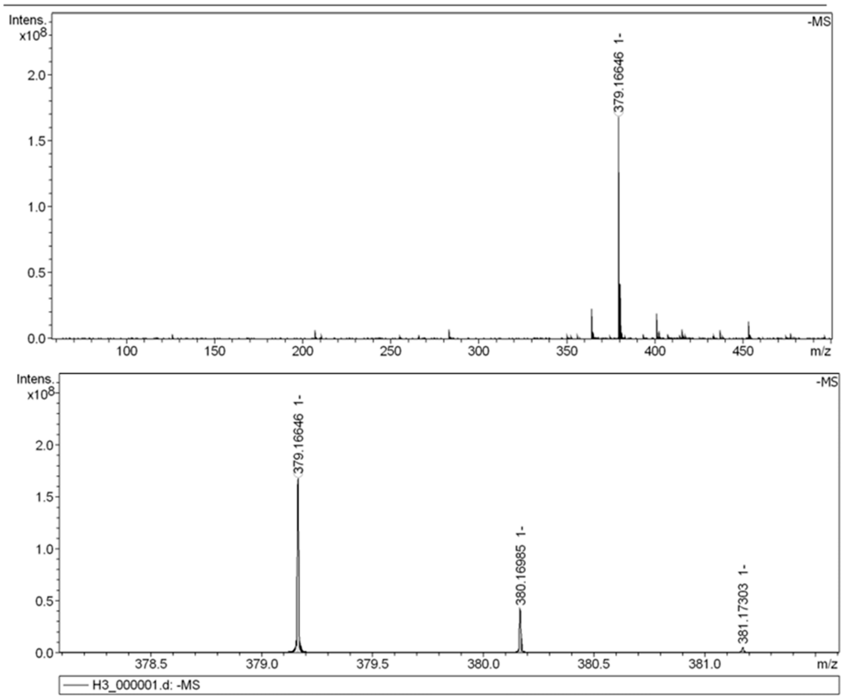


| Meas. m/z | # | Ion Formula | Score | m/z | err [ppm] | Mean err [ppm] | mSigma | rdb | e⁻ Conf | N-Rule |
|---|---|---|---|---|---|---|---|---|---|---|
| 379.166463 | 1 | C22H23N2O4 | 100.00 | 379.166331 | 0.3 | -0.5 | 5.6 | 12.5 | even | ok |

**Figure S11.** HR-MS (ESI) of H3.

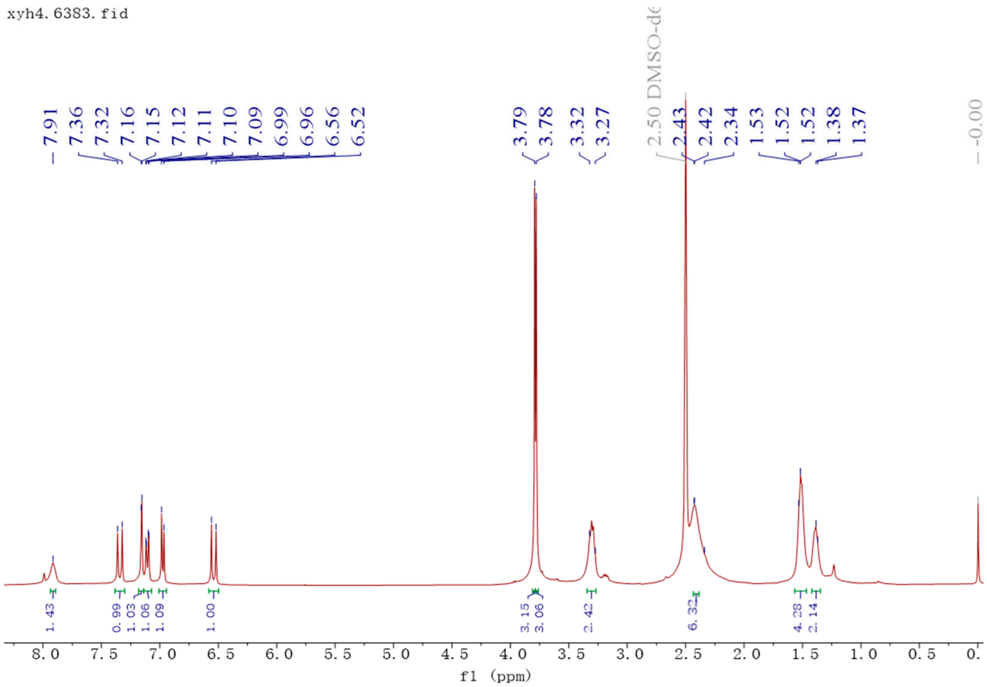


**Figure S12.** $^{1}$H NMR of H4.

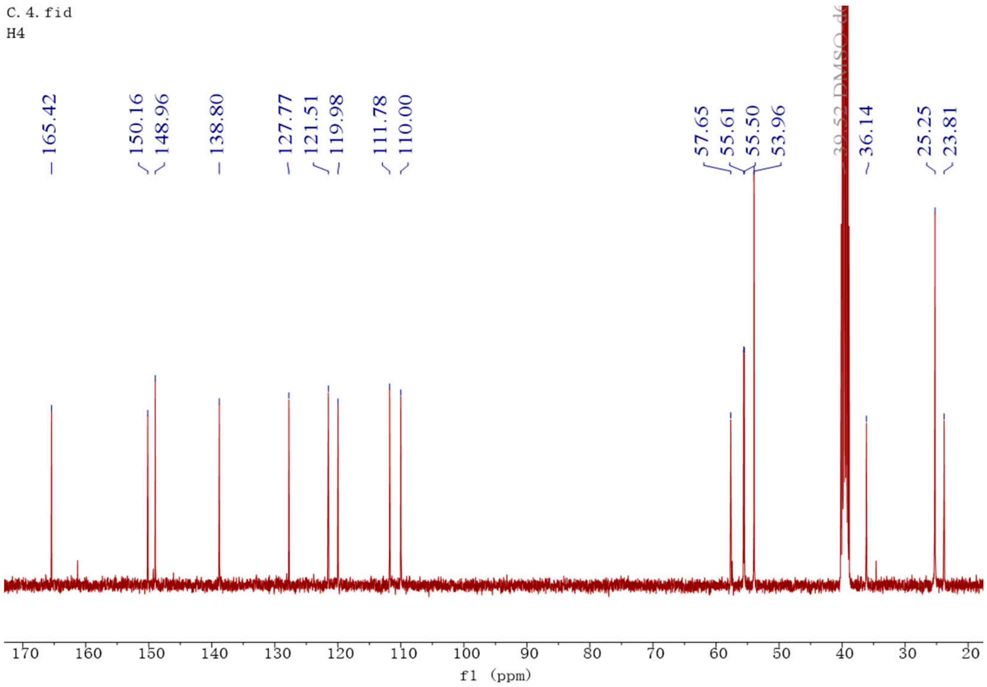


**Figure S13.** $^{13}$C NMR of H4.

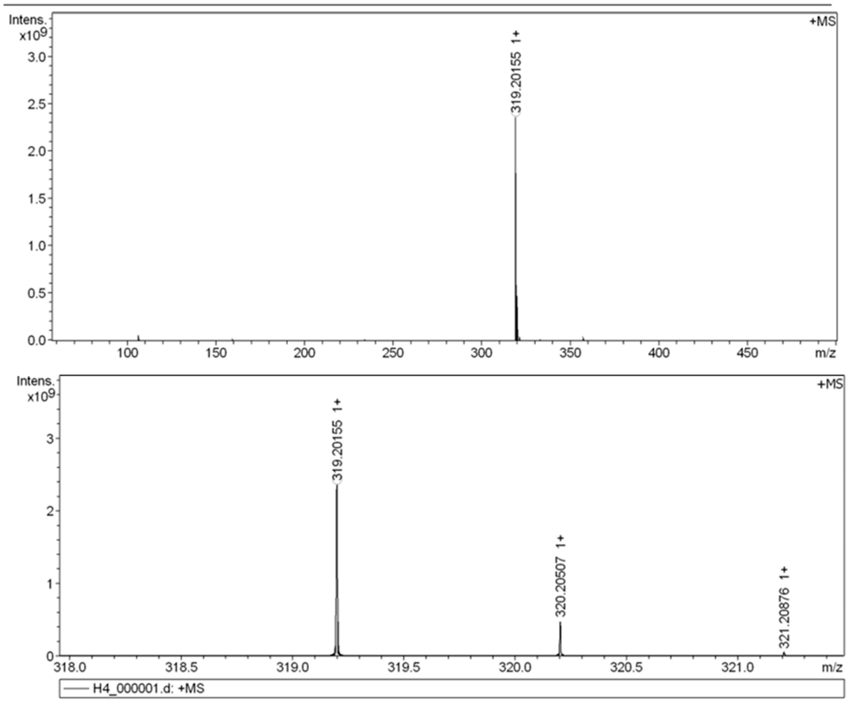


| Meas. m/z | # | Ion Formula | Score | m/z | err [ppm] | Mean err [ppm] | mSigma | rdb | e⁻ Conf | N-Rule |
|---|---|---|---|---|---|---|---|---|---|---|
| 319.201549 | 1 | C18H27N2O3 | 100.00 | 319.201619 | -0.2 | -0.0 | 5.6 | 6.5 | even | ok |

**Figure S14.** HR-MS (ESI) of H4.

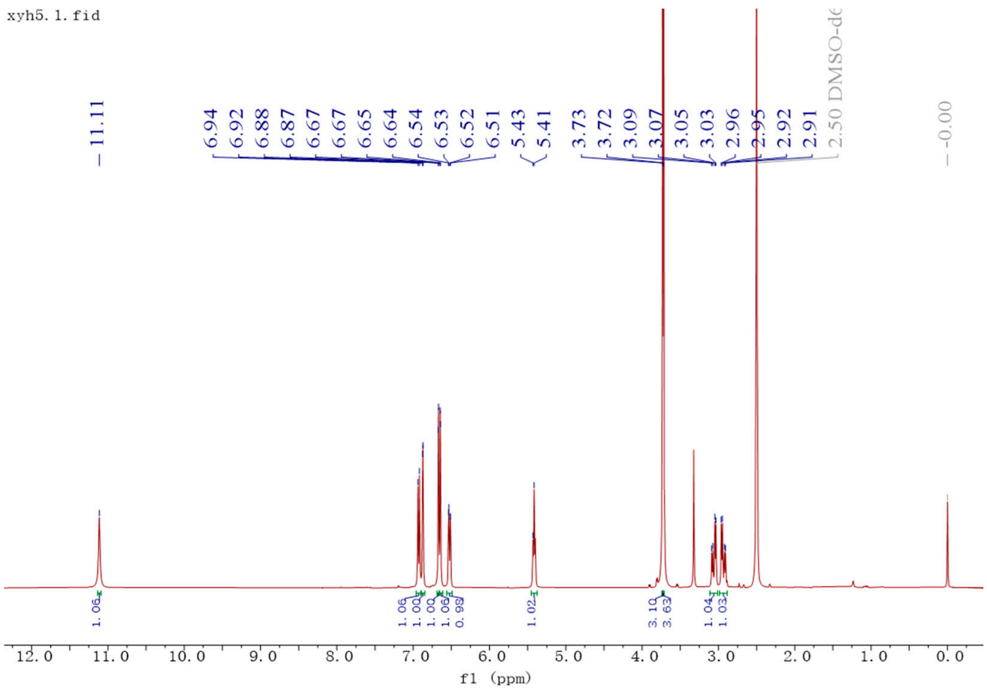


**Figure S15.** $^{1}$H NMR of H5.

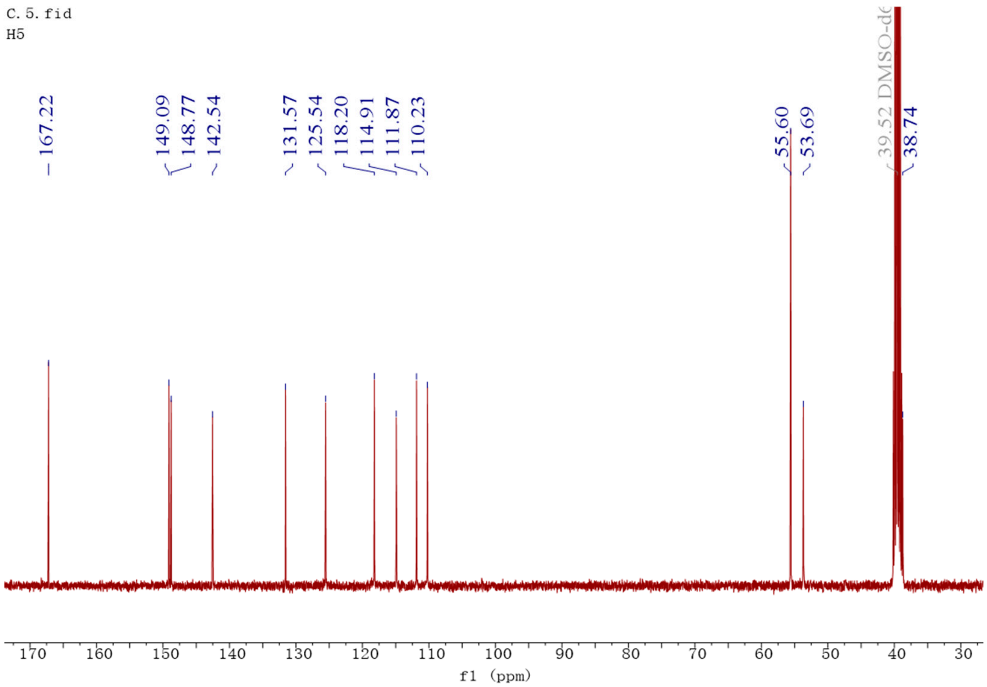


**Figure S16.** $^{13}$C NMR of H5.

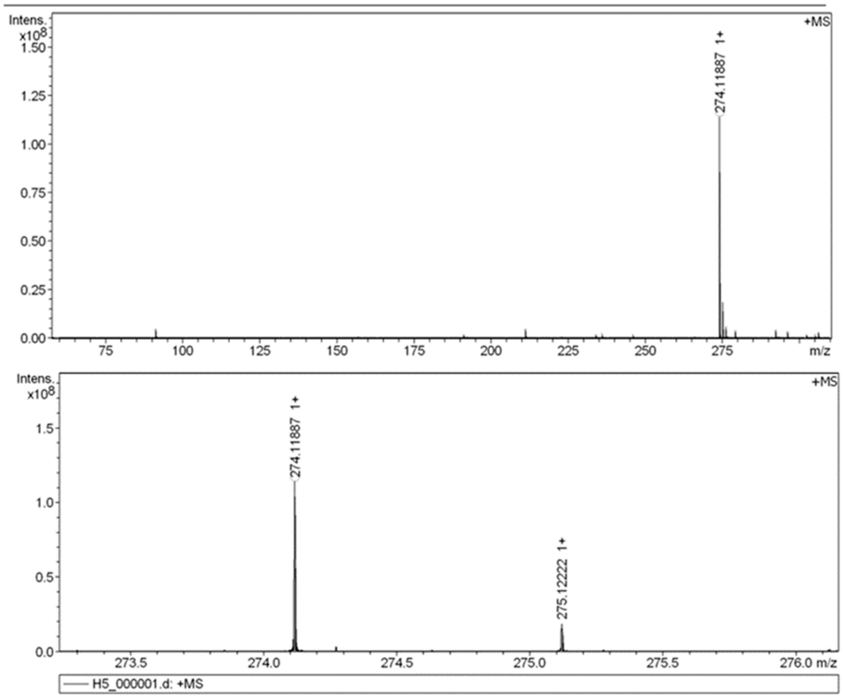


| Meas. m/z | # | Ion Formula | Score | m/z | err [ppm] | Mean err [ppm] | mSigma | rdb | e⁻ Conf | N-Rule |
|---|---|---|---|---|---|---|---|---|---|---|
| 274.118871 | 1 | C14H16N3O3 | 100.00 | 274.118618 | 0.9 | -1.2 | 4.0 | 8.5 | even | ok |

**Figure S17.** HR-MS (ESI) of H5.

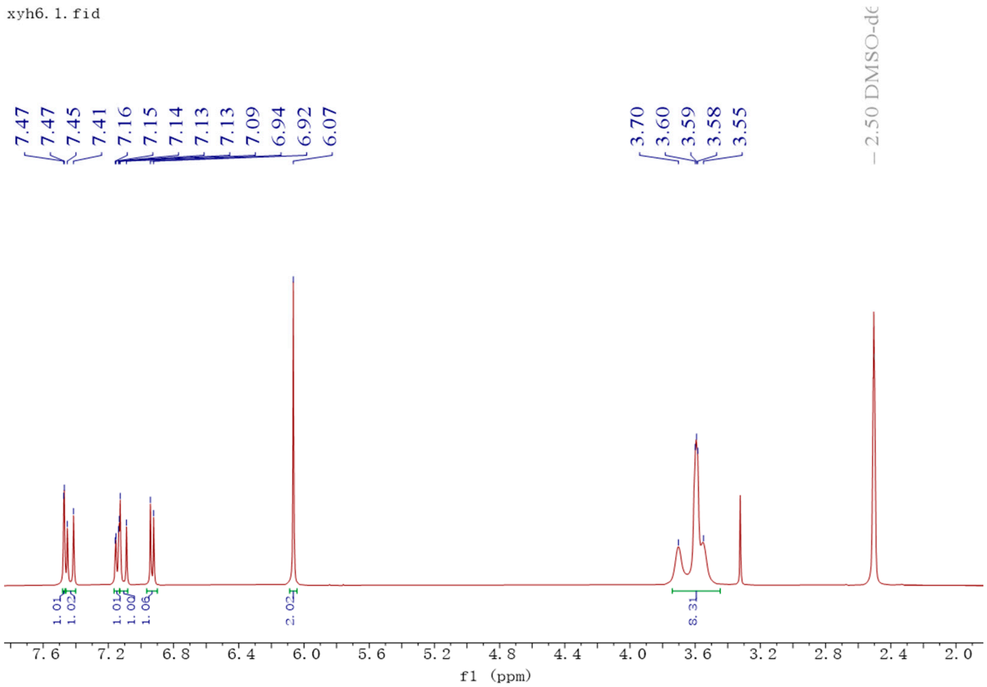


**Figure S18.** $^{1}$H NMR of H6.

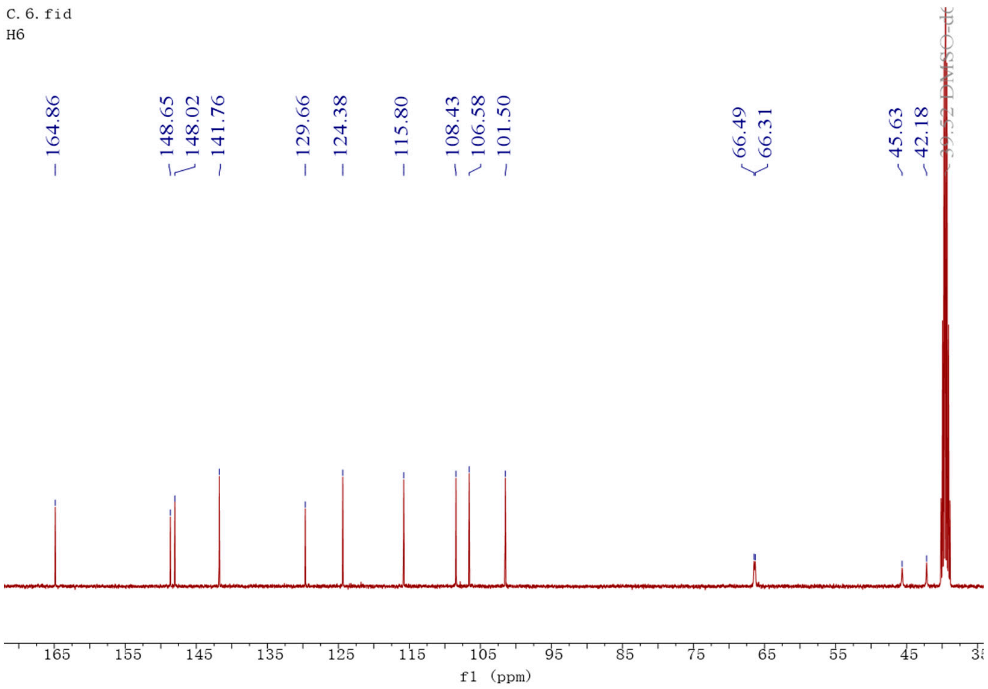


**Figure S19.** $^{13}$C NMR of H6.

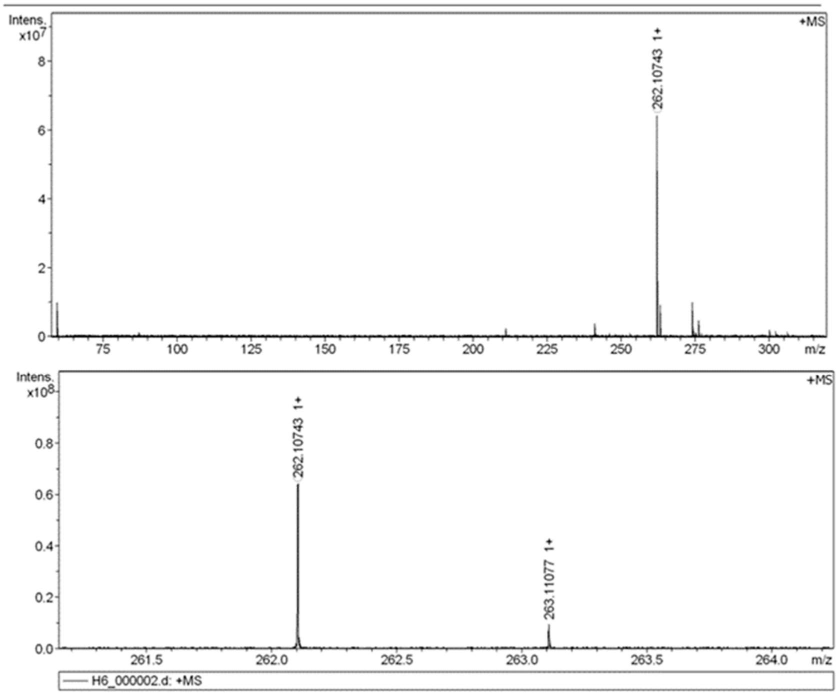


| Meas. m/z | # | Ion Formula | Score | m/z | err [ppm] | Mean err [ppm] | mSigma | rdb | e⁻ Conf | N-Rule |
|---|---|---|---|---|---|---|---|---|---|---|
| 262.107432 | 1 | C14H16NO4 | 100.00 | 262.107384 | -0.2 | -0.3 | 9.3 | 7.5 | even | ok |

**Figure S20.** HR-MS (ESI) of H6.

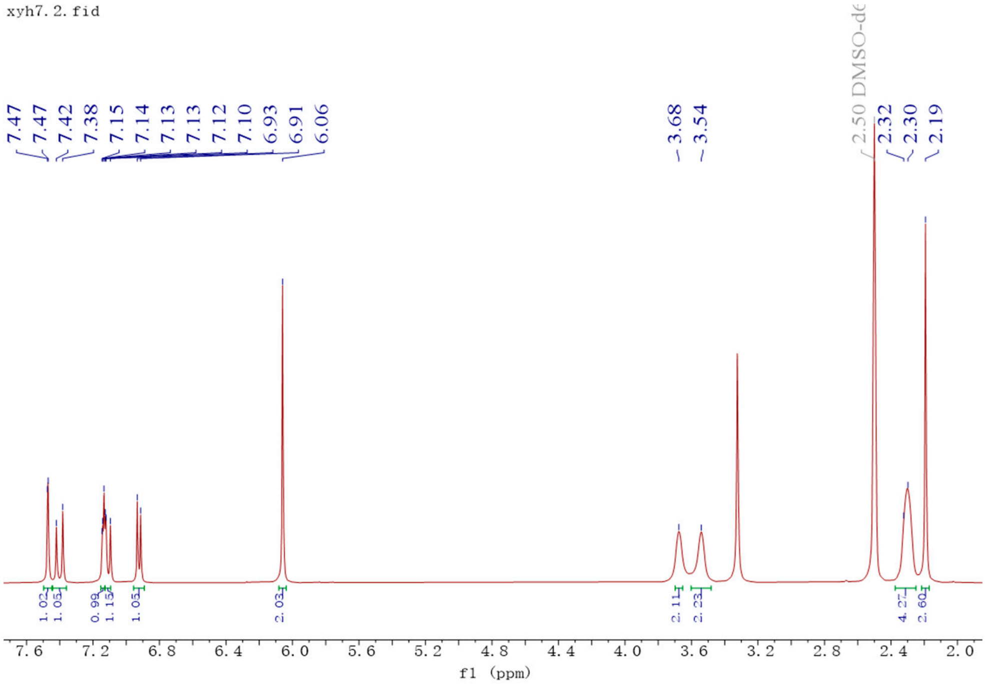


**Figure S21.** $^1$H NMR of H7.

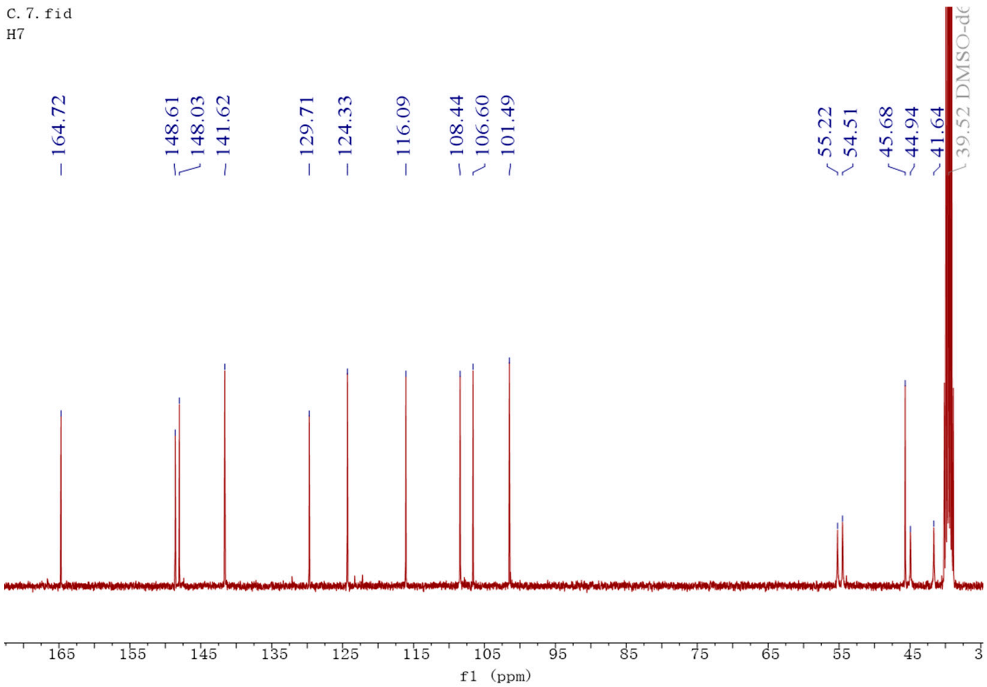


**Figure S22.** $^{13}$C NMR of H7.

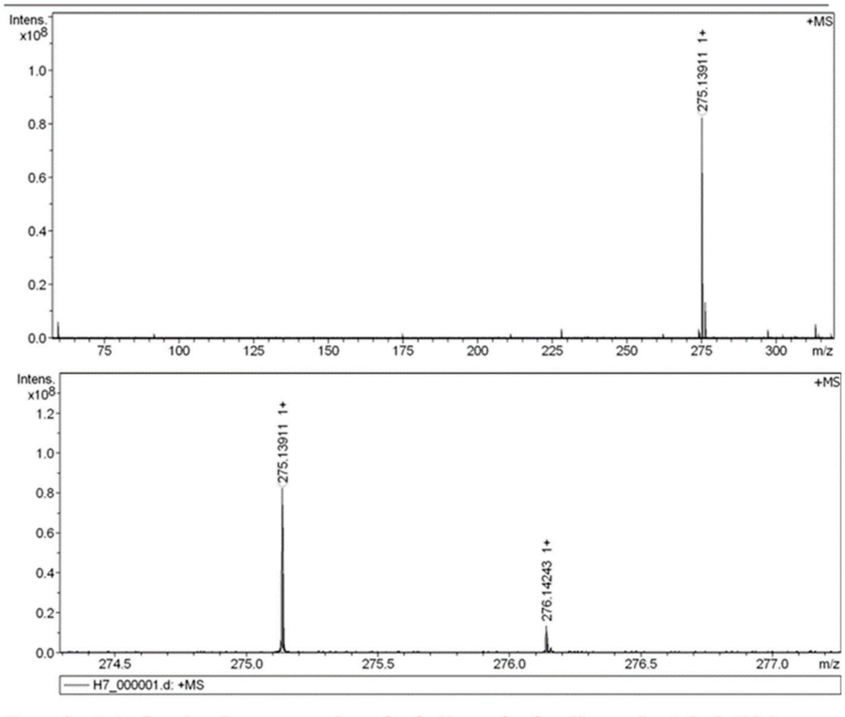


| Meas. m/z | # | Ion Formula | Score | m/z | err [ppm] | Mean err [ppm] | mSigma | rdb | e⁻ Conf | N-Rule |
|---|---|---|---|---|---|---|---|---|---|---|
| 275.139112 | 1 | C15H19N2O3 | 100.00 | 275.139019 | -0.3 | -0.5 | 7.5 | 7.5 | even | ok |

**Figure S23.** HR-MS (ESI) of H7.

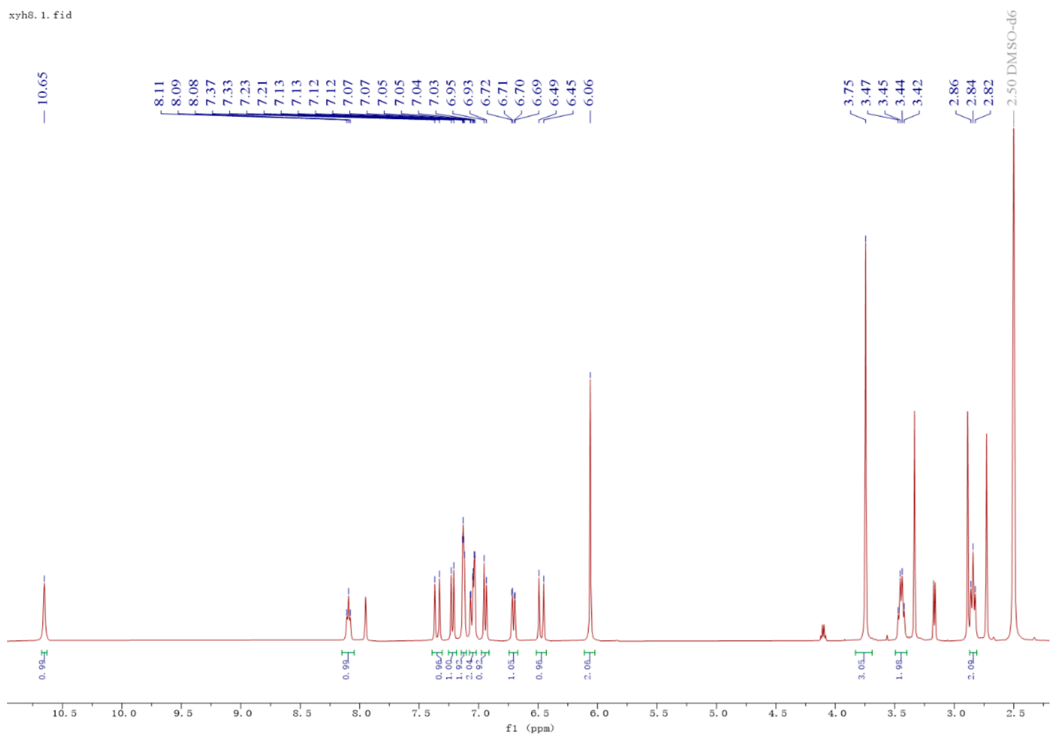

**Figure S24.** $^{1}$H NMR of H8.

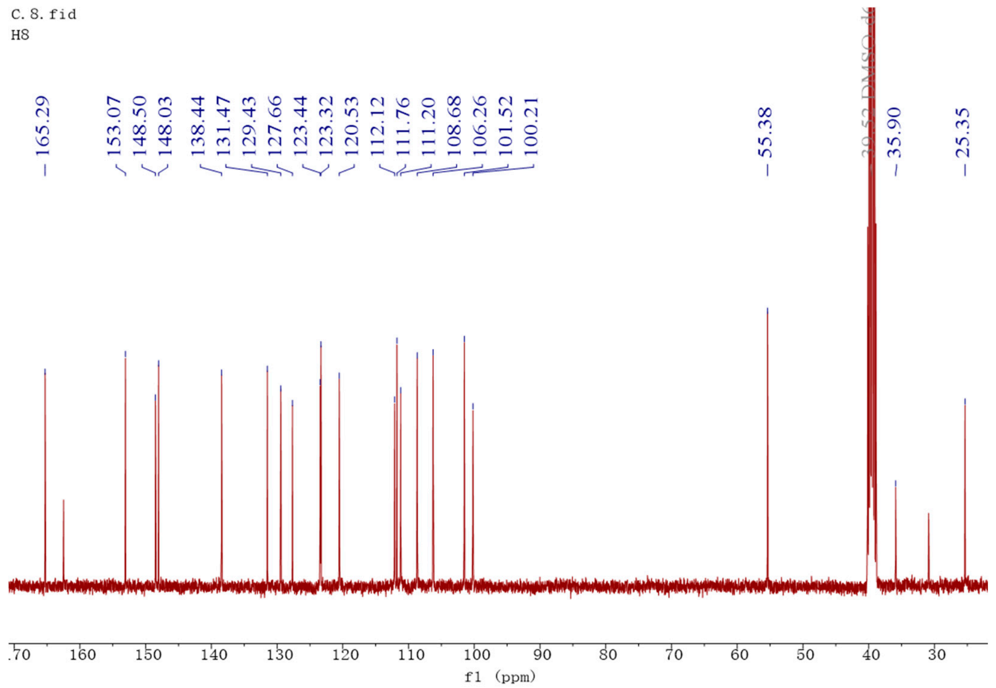

**Figure S25.** $^{13}$C NMR of H8.

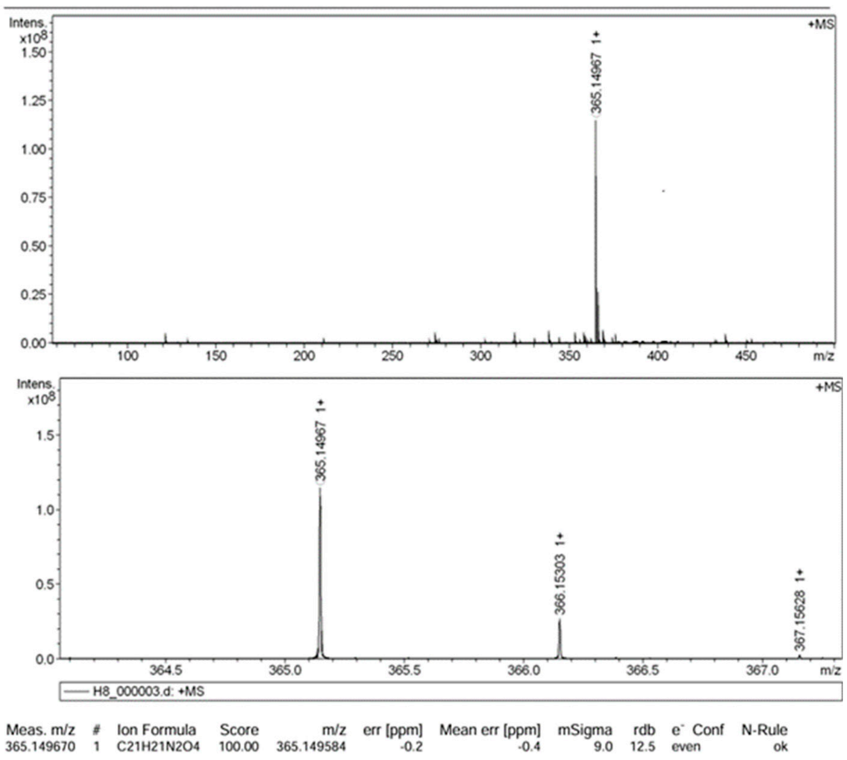


**Figure S26.** HR-MS (ESI) of H8.

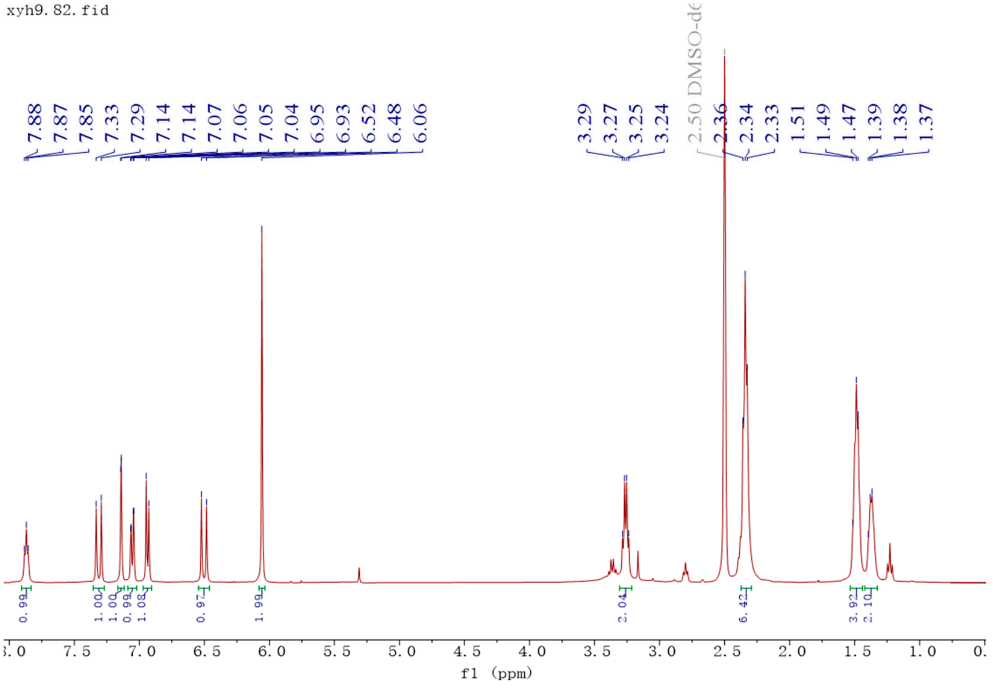


**Figure S27.** $^{1}$H NMR of H9.

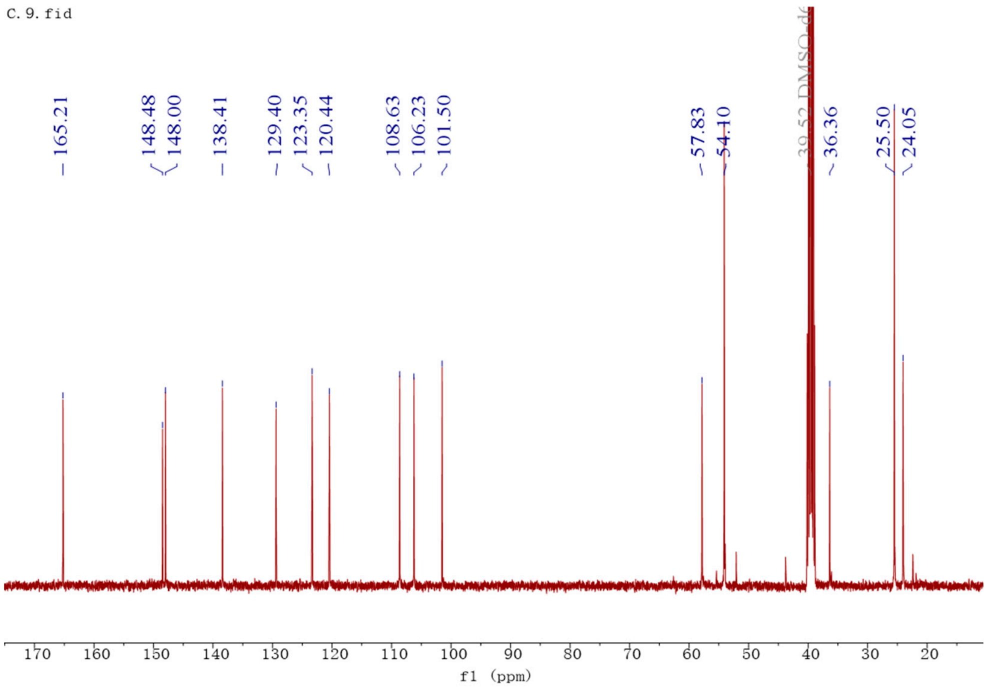


**Figure S28.** $^{13}$C NMR of H9.

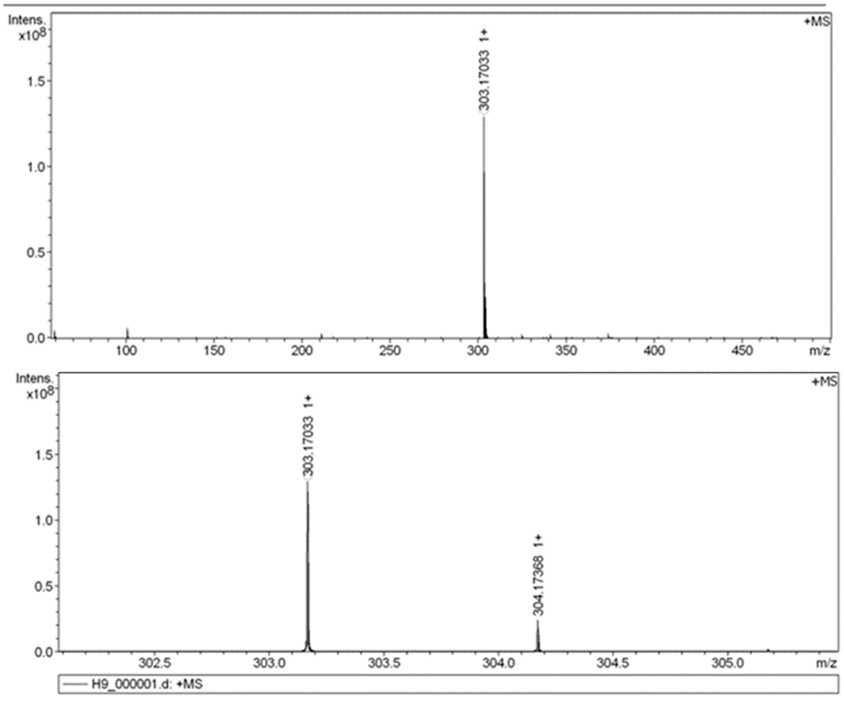


| Meas. m/z | # | Ion Formula | Score | m/z | err [ppm] | Mean err [ppm] | mSigma | rdb | e⁻ Conf | N-Rule |
|---|---|---|---|---|---|---|---|---|---|---|
| 303.170332 | 1 | C17H23N2O3 | 100.00 | 303.170319 | 0.0 | -0.2 | 8.9 | 7.5 | even | ok |

**Figure S29.** HR-MS (ESI) of H9.

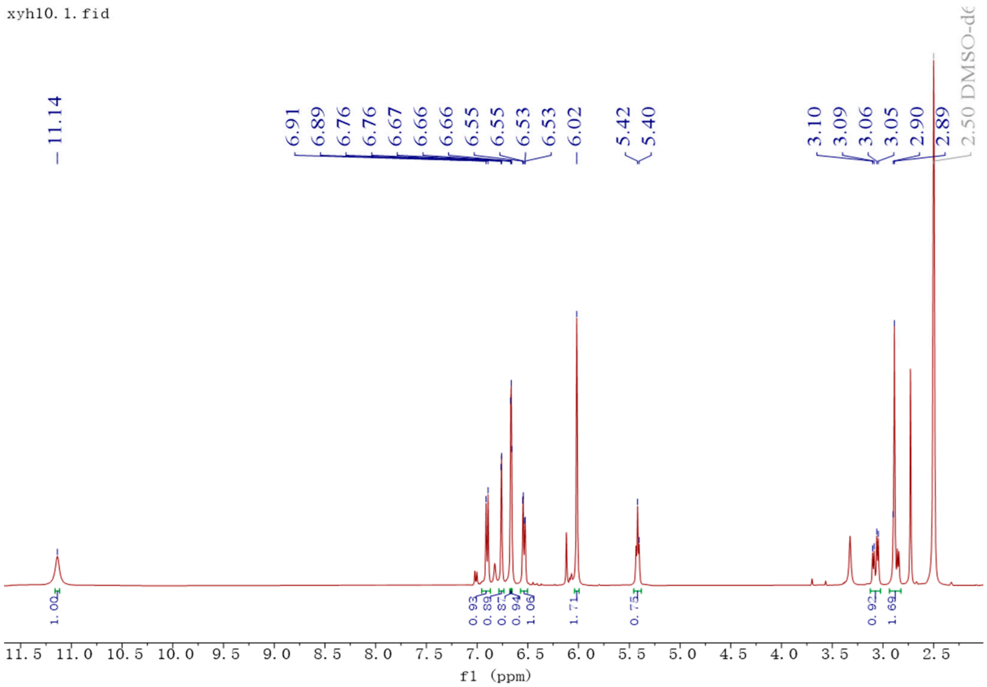


**Figure S30.** $^{1}H$ NMR of H10.

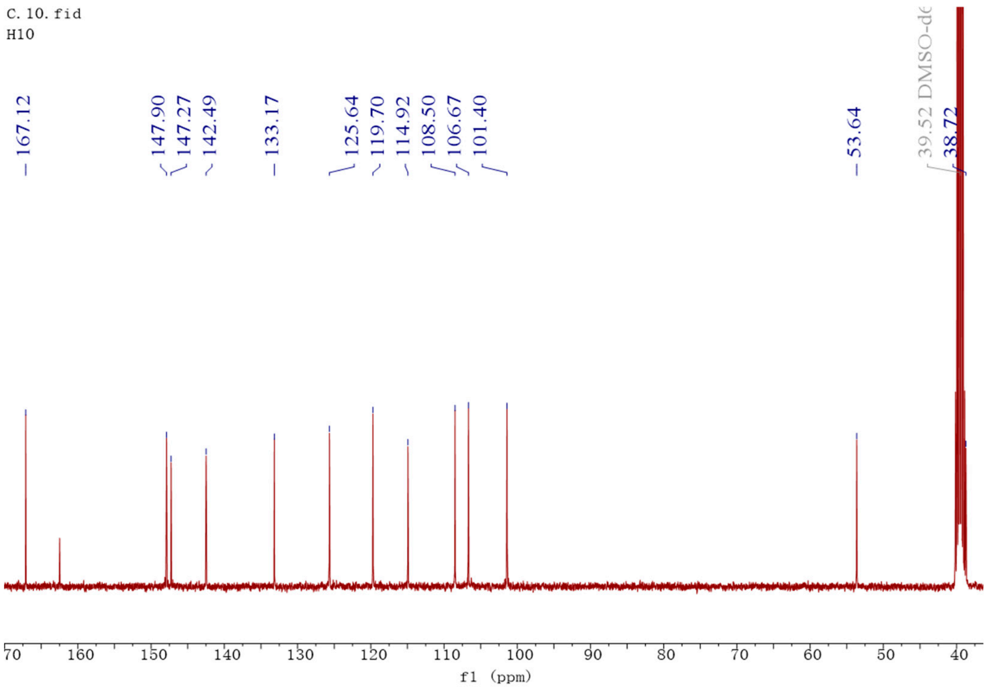


**Figure S31.** $^{13}C$ NMR of H10.

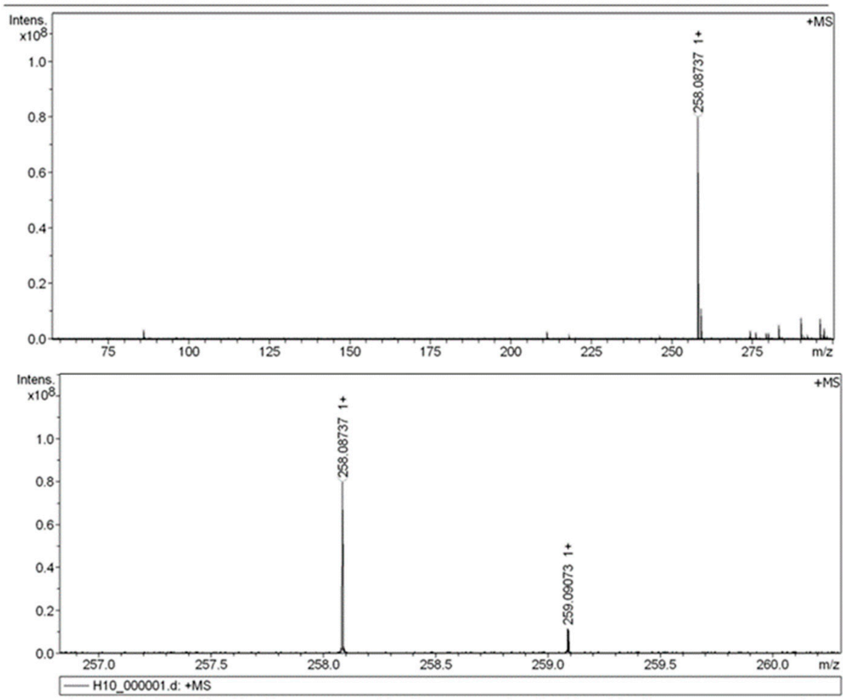


| Meas. m/z | # | Ion Formula | Score | m/z | err [ppm] | Mean err [ppm] | mSigma | rdb | e⁻ Conf | N-Rule |
|---|---|---|---|---|---|---|---|---|---|---|
| 258.087374 | 1 | C13H12N3O3 | 100.00 | 258.087318 | 0.2 | -0.5 | 10.0 | 9.5 | even | ok |

**Figure S32.** HR-MS (ESI) of H10.

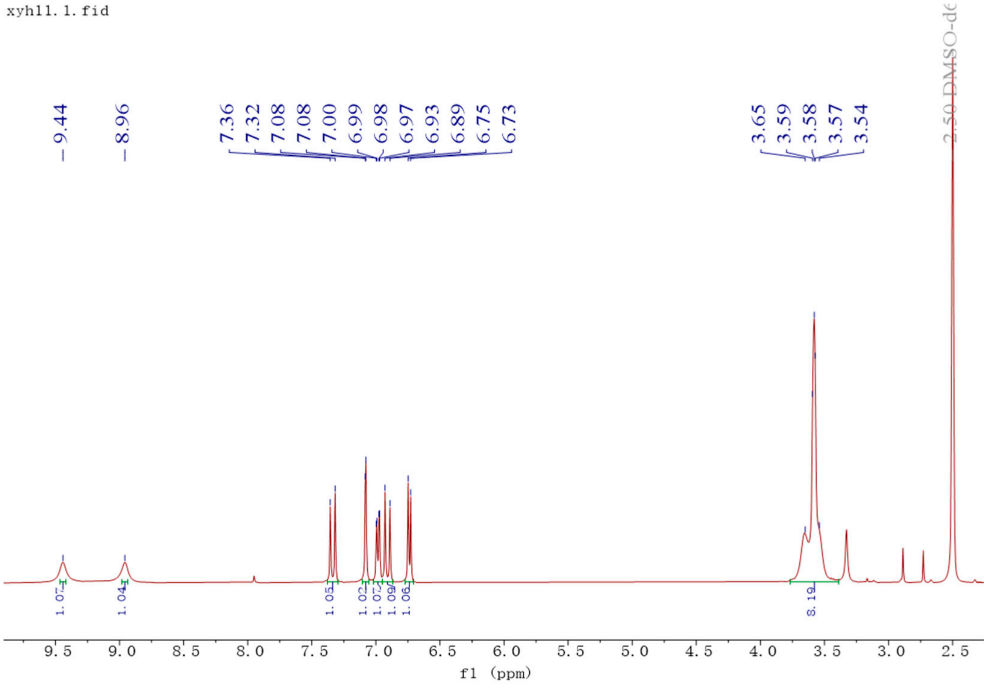


**Figure S33.** $^{1}$H NMR of H11.

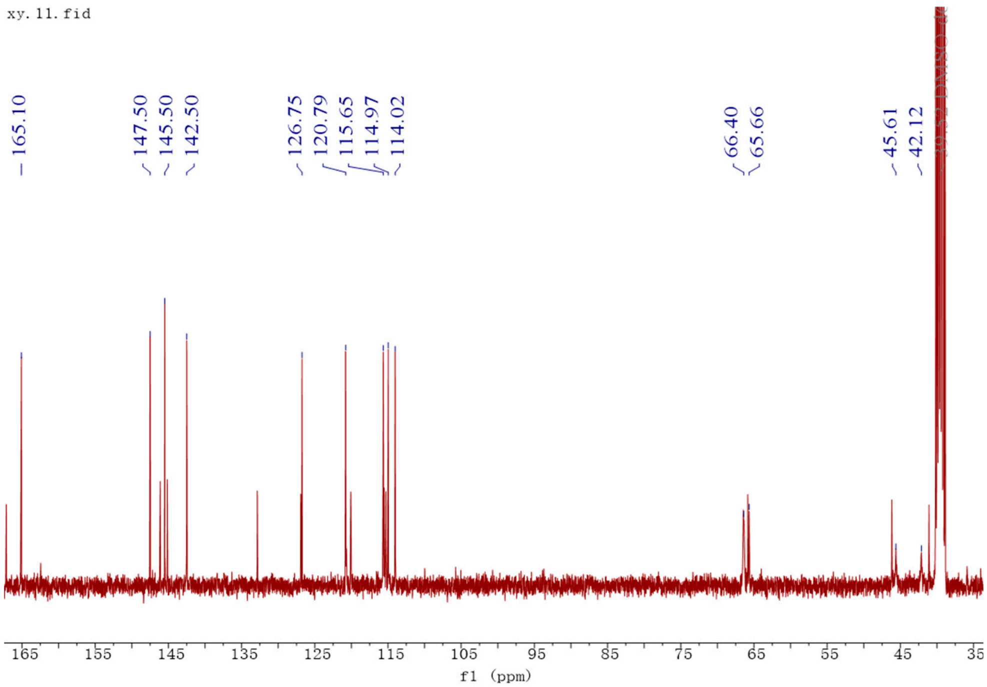


**Figure S34.** $^{13}C$ NMR of H11.

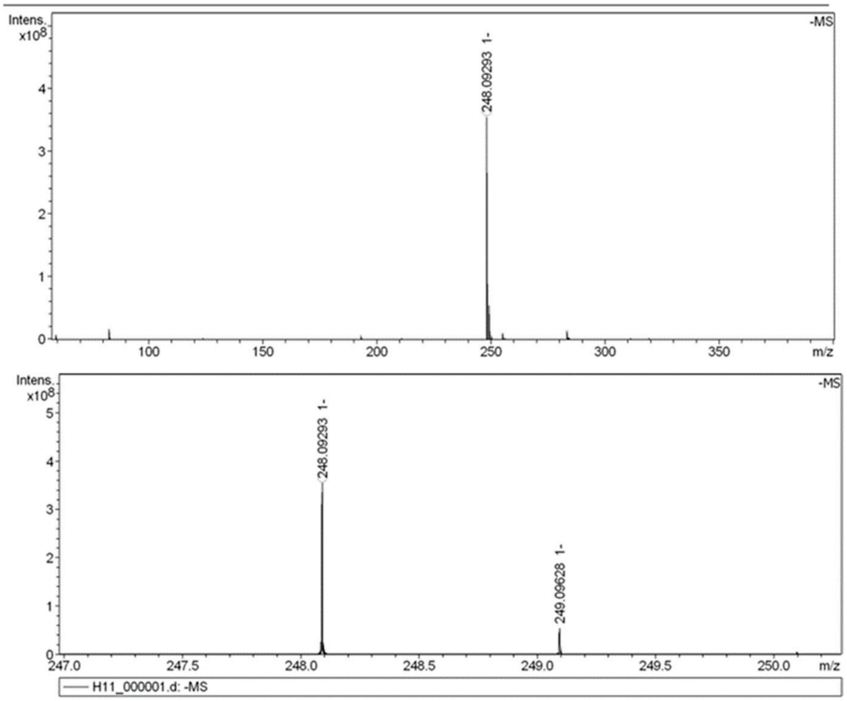


| Meas. m/z | # | Ion Formula | Score | m/z | err [ppm] | Mean err [ppm] | mSigma | rdb | e⁻ Conf | N-Rule |
|---|---|---|---|---|---|---|---|---|---|---|
| 248.092928 | 1 | C13H14NO4 | 100.00 | 248.092832 | 0.4 | -0.4 | 7.0 | 7.5 | even | ok |

**Figure S35.** HR-MS (ESI) of H11.

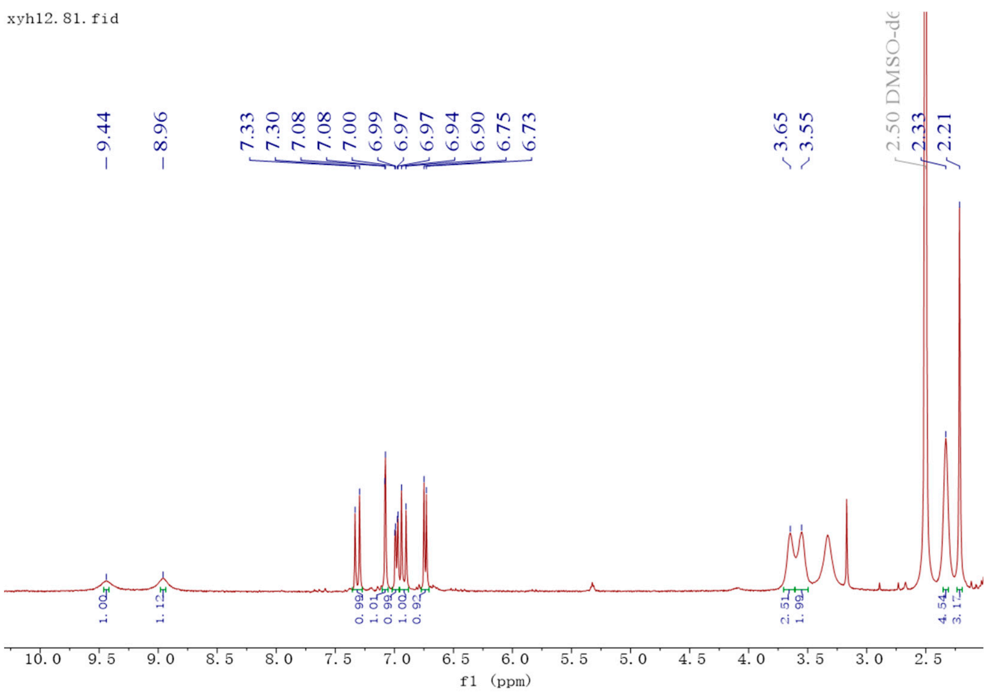


**Figure S36.** $^{1}$H NMR of H12.

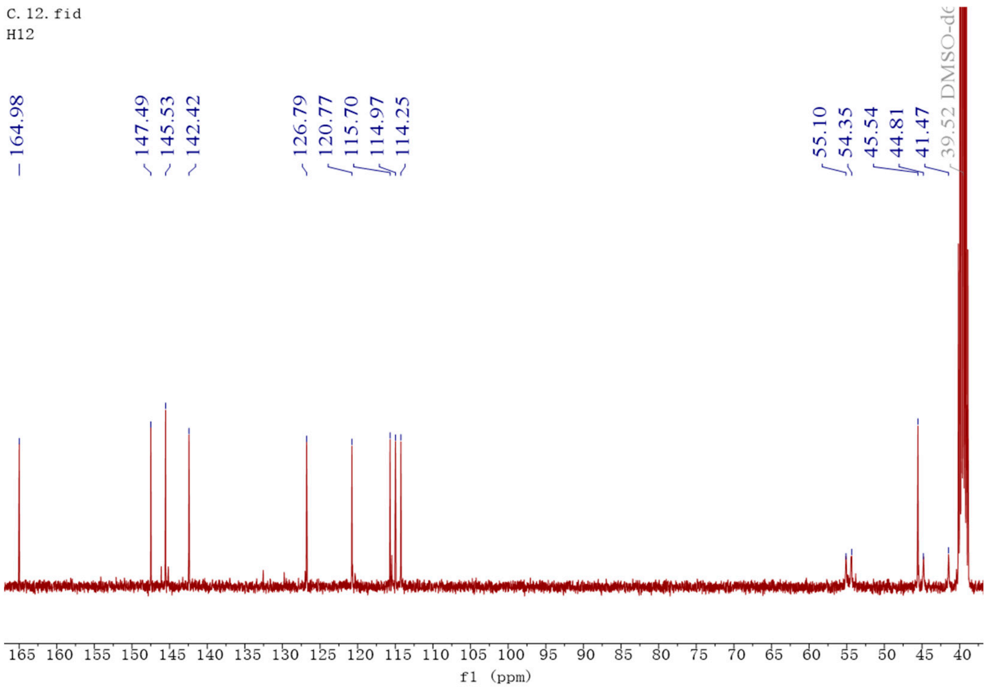


**Figure S37.** $^{13}$C NMR of H12.

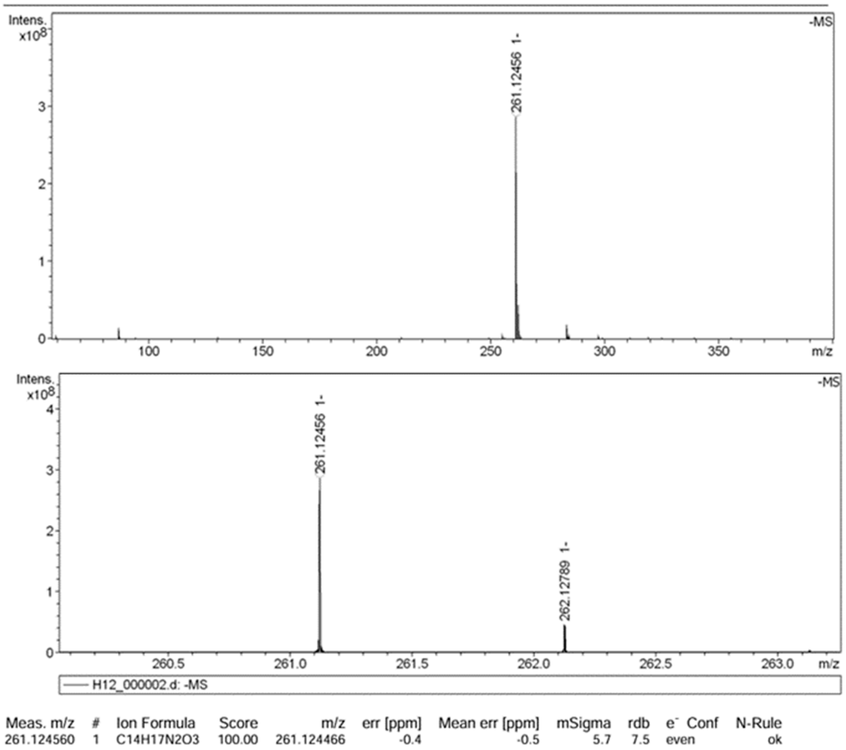

**Figure S38.** HR-MS (ESI) of H12.

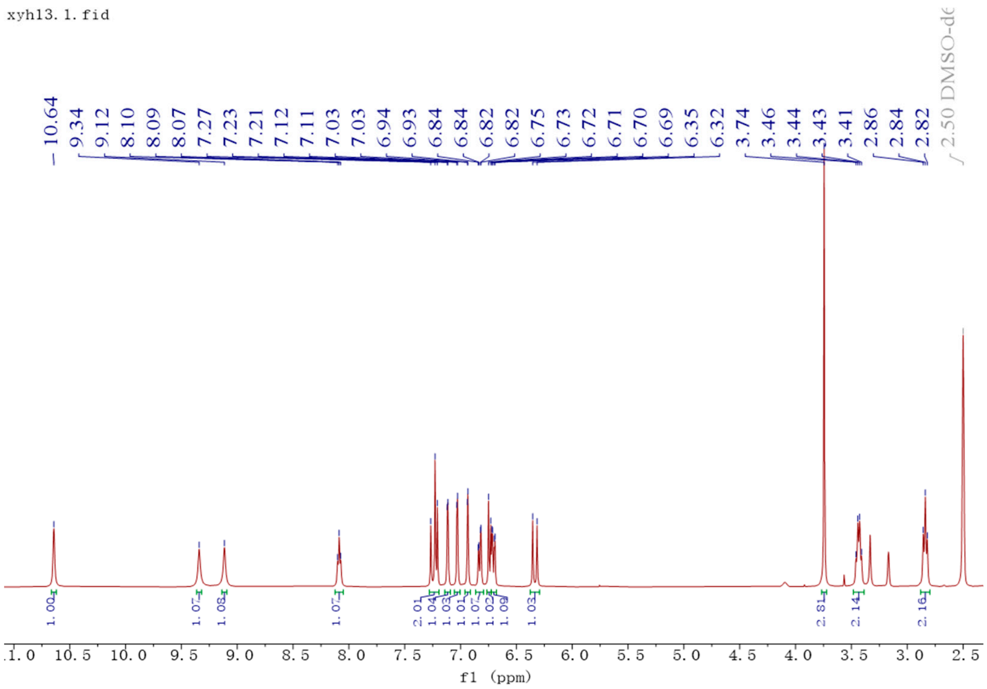

**Figure S39.** $^{1}$H NMR of H13.

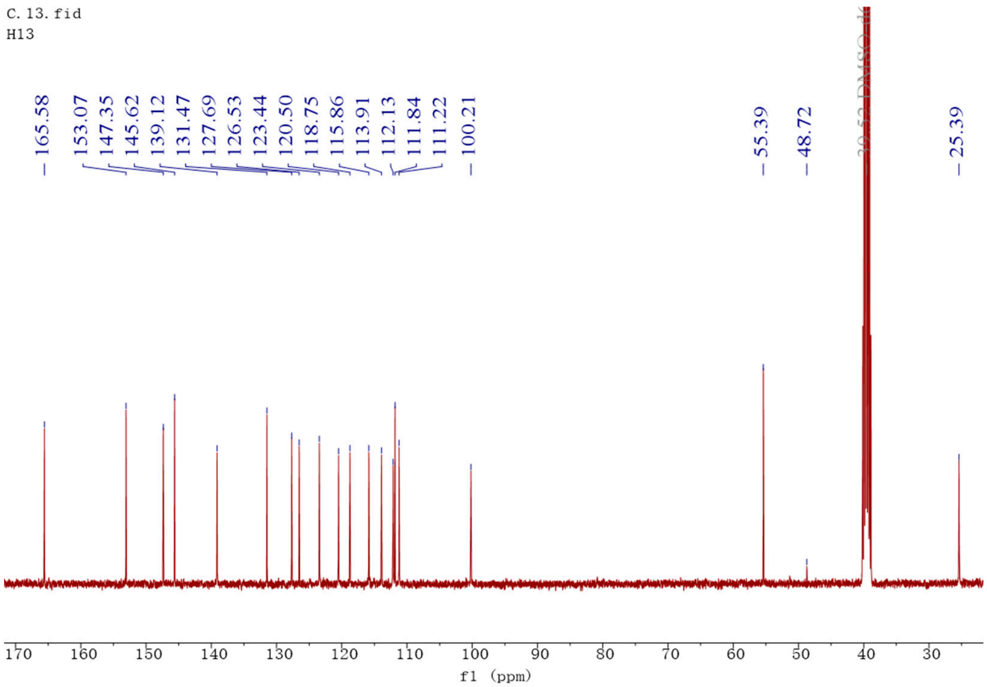


**Figure S40.** $^{13}$C NMR of H13.

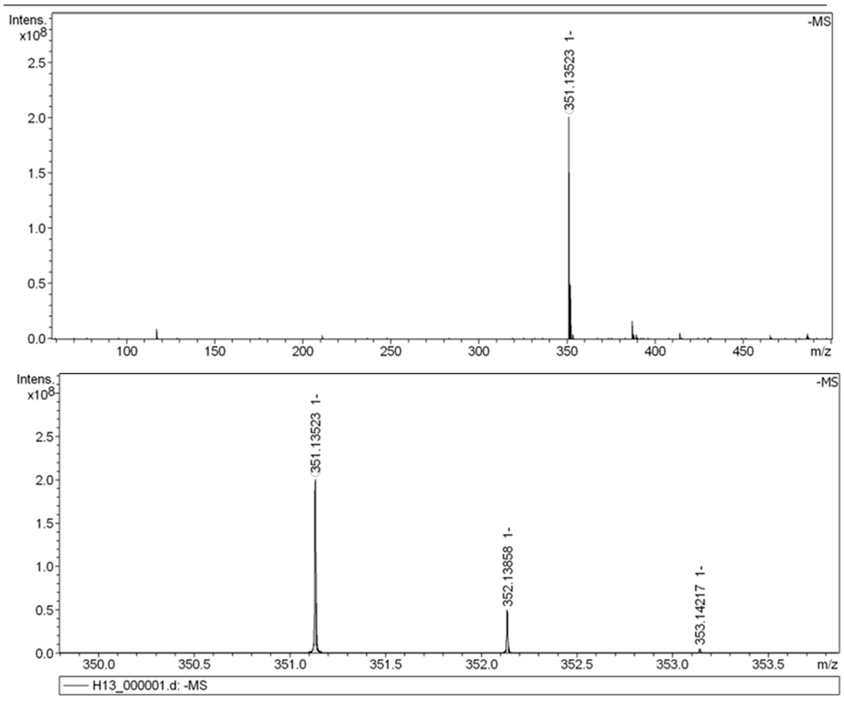


| Meas. m/z | # | Ion Formula | Score | m/z | err [ppm] | Mean err [ppm] | mSigma | rdb | e⁻ Conf | N-Rule |
|---|---|---|---|---|---|---|---|---|---|---|
| 351.135226 | 1 | C20H19N2O4 | 100.00 | 351.135031 | 0.6 | -0.7 | 8.2 | 12.5 | even | ok |

**Figure S41.** HR-MS (ESI) of H13.

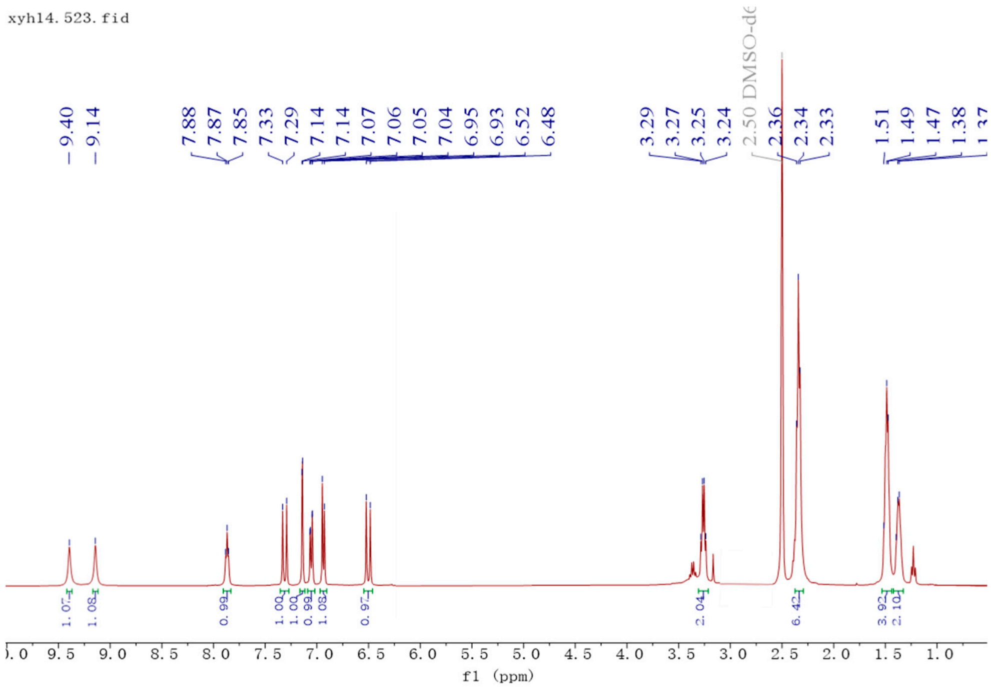

**Figure S42.** $^{1}H$ NMR of H14.

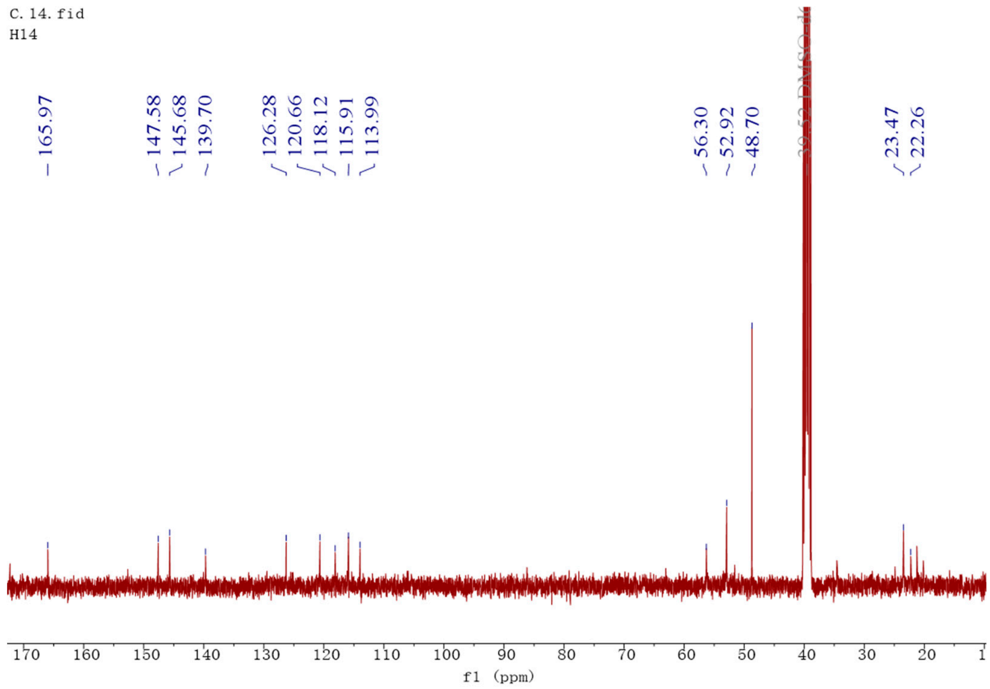

**Figure S43.** $^{13}C$ NMR of H14.

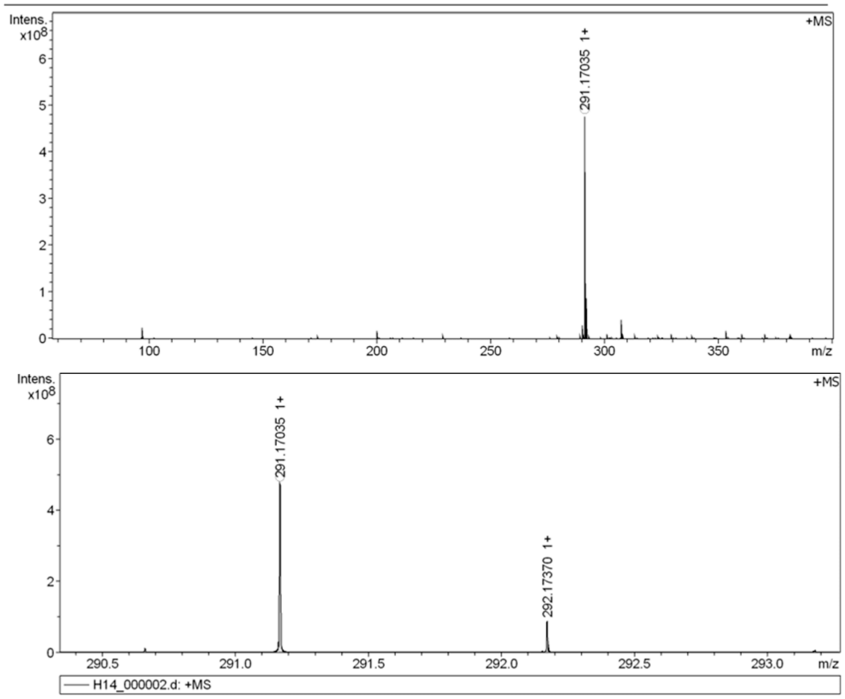


| Meas. m/z | # | Ion Formula | Score | m/z | err [ppm] | Mean err [ppm] | mSigma | rdb | e⁻ Conf | N-Rule |
|---|---|---|---|---|---|---|---|---|---|---|
| 291.170346 | 1 | C16H23N2O3 | 100.00 | 291.170319 | -0.1 | -0.3 | 5.9 | 6.5 | even | ok |

**Figure S44.** HR-MS (ESI) of H14.
